# Immediate and Sustained Effects of Intensive Equine-Assisted Physiotherapy Based on Neuroproprioceptive “Facilitation and Inhibition” on Psychomotor Development, Clinical Functions, Quality of Life, and Molecular Biological Indicators in Children With Spinal Muscular Atrophy: Protocol for a Crossover Randomized Controlled Trial

**DOI:** 10.2196/83266

**Published:** 2026-02-19

**Authors:** Katerina Marikova, Jindra Reissigova, Miloslav Vilimek, Marie Cerna, Marketa Pokorna, Kamila Rasova

**Affiliations:** 1 Department of Rehabilitation, Third Faculty of Medicine, Charles University and University Hospital Kralovske Vinohrady, Ruská 87, Prague, 100 00, Czech Republic, 420604511416; 2 The Czech Academy of Sciences, Institute of Computer Science, Prague, Czech Republic; 3 College of Polytechnics Jihlava, Jihlava, Czech Republic; 4 Department of Medical Genetics, Third Faculty of Medicine, Charles University, Prague, Czech Republic

**Keywords:** spinal muscular atrophy, genetic therapy, equine-assisted therapy, child, pediatrics, physical therapy modalities, long noncoding RNA, neuroproprioceptive facilitation, neuroproprioceptive inhibition, The Children’s Hospital of Philadelphia Infant Test of Neuromuscular Disorders

## Abstract

**Background:**

Spinal muscular atrophy (SMA) is a rare neuromuscular disease and the most common genetic cause of infant death. Although pharmacological treatment improves survival rates and functional capacity, physiotherapy remains a key component of care. A newly developed innovative equine-assisted physiotherapy method based on neuroproprioceptive “facilitation and inhibition” principles (NEUROEQUIP-SMA) is hypothesized to improve the quality and extent of motor development in children with SMA.

**Objective:**

The aim of this study is to assess the efficacy of NEUROEQUIP-SMA compared with standard individual outpatient physiotherapy based on the same neuroproprioceptive “facilitation and inhibition” principles (SMA-SOC-N) and to evaluate its effects on functional outcomes and quality of life (QoL). In addition, the response of molecular biomarkers to treatment will be assessed.

**Methods:**

In this crossover randomized controlled trial, 20 children with SMA types I-III, aged 2‐9 years, will participate in two 6-day therapy programs (A and B) of equal duration (50 min per day) and separated by a 10-week washout period. Each child will be randomly assigned to receive the therapies in either the AB or BA sequence. Therapy A consists of a newly developed NEUROEQUIP-SMA (15 min twice daily), whereas therapy B involves SMA-SOC-N (30 min once daily). Both programs include therapeutic horse grooming (20 min a day). The Children’s Hospital of Philadelphia Infant Test of Neuromuscular Disorders (CHOP INTEND) was selected as the primary outcome measure. Secondary outcomes included motor coordination assessed through 3D motion analysis, muscle fatigue, spirometry, and standardized clinical tests and rating scales, as well as monitoring of psychomotor development (via home-video analysis) and QoL (via questionnaires). Molecular biomarkers will be analyzed from blood samples. The immediate effect of the intervention will be evaluated for most outcomes, while psychomotor development and QoL will be monitored 28 days after therapy as longer-term outcomes. Treatment effect sizes will be reported alongside *P* values to illustrate the magnitude of changes in the outcomes. The study was approved by the Ethics Committee of the Third Faculty of Medicine, Charles University, under the number UK3LF/658559/12025.

**Results:**

The study is designed for 20 participants. Data collection will begin in February 2026 and will be completed in May 2026. Data analyses are planned for autumn 2026, and study results are expected to be available in 2027. A paired *t* test comparing the primary outcome (CHOP INTEND) between treatments NEUROEQUIP-SMA and SMA-SOC-N in 20 children will have 80% power to detect moderate-to-large effect size (Cohen *d*=0.66) at a 5% significance level.

**Conclusions:**

This trial will be the first study to evaluate the effects of NEUROEQUIP-SMA in children with SMA. If preliminary findings confirm a benefit, this physiotherapy approach may represent a promising adjunct to care for the generation of children undergoing gene therapy.

## Introduction

### Background

Spinal muscular atrophy (SMA) is an autosomal recessive neuromuscular disorder caused by the loss or mutation of the *SMN1* gene, with retention of its paralog *SMN2*. Despite the latest treatment options, it is the leading genetic cause of infant mortality under 2 years of age and affects approximately 1 in 10,000 live births in the Czech Republic [1].

The primary characteristic of SMA is the degeneration of alpha motor neurons in the anterior horn of the spinal cord [2]. This leads to a wide range of clinical manifestations, from severe neonatal weakness and respiratory failure to mild proximal weakness presenting in adulthood [3,4]. Although new pharmacological advancements are altering SMA phenotypes and subsequently the clinical manifestation of the disease [5,6], multidisciplinary supportive care remains essential to achieve optimal outcomes [2,7], with physiotherapy being an integral part of it.

The perspective on physiotherapy has been rapidly evolving in recent years. In the past, it was prohibited, as it was assumed that it might accelerate the degeneration of diseased muscles [8,9]. This noninterventionist approach proved to be ineffective, as it deepened muscle weakness and fatigue, leading to mobility impairments [2,10]. Over time, interventions such as positioning, breathing exercises, range-of-motion activities, and targeted strengthening began to be used to prevent complications (eg, contractures and deformities). Nowadays, the focus has shifted more toward enhancing functional mobility, using techniques such as balance training, developmental activities, and gait rehabilitation [11-14].

Experimental research [15] showed a close link between neuron-specific protection and their activation levels by different exercise parameters. Exercise-induced neuroprotection was independent of SMN protein expression and was associated with specific metabolic and behavioral adaptations, including a reduction in muscle fatigability. This new insight into the motor units’ adaptations significantly influenced and motivated us to develop a new active physiotherapy approach for children with SMA. We decided to use the principles of neuroproprioceptive “facilitation and inhibition” in therapy, in which it is assumed that, through the combination of appropriate afferent stimuli, the interneuronal system is activated, allowing the transmission of missing signals and enabling the activation of movements that the patient would otherwise be unable to consciously perform. This approach is used in the Czech Republic for neurologically impaired individuals, such as poststroke individuals [16] or people with multiple sclerosis [17]. However, it has not yet been studied in children with SMA.

We have developed a unique method of equine-assisted physiotherapy based on neuroproprioceptive “facilitation and inhibition” (NEUROEQUIP-SMA). Standard Equine-Assisted Physiotherapy has the potential to influence multiple domains, including posture, balance, and coordination, through the dynamic and repetitive movement provided by the horse [18-20]. It has been demonstrated that, in children with cerebral palsy, it shows benefits such as improved postural control and reduced spasticity [18,21,22]. In children with SMA [23], it leads to physical improvements (enhanced muscle strength and better balance) and psychological improvements (self-confidence, self-esteem, pride, independence, and a sense of achievement).

This study primarily aims to assess the effectiveness of 2 physiotherapy approaches: NEUROEQUIP-SMA and a standard physiotherapy protocol based on neuroproprioceptive “facilitation and inhibition” principles (SMA-SOC-N). We hypothesize that NEUROEQUIP-SMA will produce greater improvements in functional and postural outcomes in children with SMA due to:

1. The rider moving in harmony with the horse’s motion rather than resisting it as in conventional stabilization techniques.
2. Simultaneous activation of multiple muscle chains, enabling coordinated engagement of weakened muscles.
3. Afferent input combination that initiates movement from the pelvis, lowers the center of gravity, supports the diaphragm in an expiratory position, relaxes the shoulder girdle, and improves overall stability.
4. Increased sensory input intensity and greater movement complexity, further promoting neuromuscular activation.

### Primary Hypothesis

Immediately after the 6-day intensive program, we expect improvement in both groups in the Children’s Hospital of Philadelphia Infant Test of Neuromuscular Disorders (CHOP INTEND); however, we anticipate more pronounced effects in the NEUROEQUIP-SMA group.

### Secondary Hypotheses

We anticipate improvements in both groups; however, more pronounced effects are expected in the NEUROEQUIP-SMA group. Specifically, we hypothesize:

1. Reduced undesirable muscle fatigue, assessed using surface electromyography (sEMG) immediately after completion of the program.
2. Improved coordination of the torso and cervical spine, enhanced weight-shift control, and optimized diaphragmatic breathing in the abdominal region immediately after completion of the program.
3. Stabilized or improved motor function, as measured by the Motor Function Measure–20 (MFM-20), immediately after completion of the program.
4. Improved respiratory function and spinal alignment, reflected in spirometry and scoliometric outcomes immediately after completion of the program.
5. Improved trunk control and selective motor control, as demonstrated by performance in the Segmental Assessment of Trunk Control (SATCo), Trunk Control Measurement Scale (TCMS), Modified Pediatric Reach Test, and Selective Control Assessment of the Lower Extremity (SCALE) immediately after completion of the program.
6. Enhanced quality of life assessed using the International Classification of Functioning, Disability and Health (ICF)-based Documentation Form for the Brief Category of Children with Cerebral Palsy, the Strengths and Difficulties Questionnaire, and the Pediatric Quality of Life Inventory 4.0 Generic Core Scales, 28 days after completion of the program.
7. Improved quality and range of psychomotor development, evaluated through home video recordings conducted 28 days after completion of the program.

### The Monitoring of Neurophysiological Mechanisms of Therapy Effects

As a complementary and innovative approach to document the benefits of equine-related activities, monitoring will be complemented by analysis of long noncoding RNAs (lncRNAs) expression in peripheral blood. These 200+ nucleotide long transcripts (eg, SMN-AS1) are involved in the regulation of gene expression, for example, through association with polycomb repressive complex 2 [24]. Their deregulated levels are associated with many pathological conditions including neurodegenerative diseases. Changes in the expression of selected lncRNAs in body fluids may also reflect changes in the body in response to therapeutic intervention [25]. We propose a set of selected lncRNAs as a biomarker of improvement status in SMA patients after the inclusion of intensive therapy. Based on previous studies, we selected the lncRNAs (MALAT1 and PARTICLE), which interact with PCR2 [26]. This makes them similar to SMN-AS1, which inhibits SMN2 expression by binding PCR2 [24]. MEG3, MALAT1, and NEAT1 are lncRNAs involved in motor neuron development [27], which are affected in this disease. Deregulated levels of the lncRNAs, GAS5, and H19 have been detected in the blood of patients with neurodegenerative disease [25]. Analysis of selected lncRNAs in the blood of SMA patients before and after intensive therapy could provide novel diagnostic and prognostic biomarkers and therapeutic targets and allow for an understanding of the neurophysiological effects of the treatment. It is anticipated that measurable alterations in lncRNA expression will be detectable immediately following completion of the 6-day therapeutic program.

The aim of this protocol is to present the design and procedures of the randomized crossover study and to specify how the effectiveness of a unique method of equine-assisted physiotherapy based on neuroproprioceptive “facilitation and inhibition” will be tested against standard physiotherapy based on the same principles in children with SMA.

## Methods

### Study Design

This study is designed as a single-blind randomized crossover-controlled trial. The protocol was substantially revised after the first peer review round, and the trial was newly registered on ClinicalTrials.gov on November 11, 2025 (Unique Protocol ID: 4424; NCT07336602). Participants will be children with SMA types I, II, and III, aged 2 to 9 years. Every child will undergo two 6-day therapeutic programs, administered in 2 separate periods. During each period, children receive 30 minutes of individual treatment and 20 minutes of therapeutic horse grooming per day. In total, 2 different concepts (A and B) will be used for the individual therapy. Therapy A is a newly developed NEUROEQUIP-SMA, while therapy B, the active comparator, is SMA-SOC-N (Table 1). Participants will be randomized in a 1:1 ratio to receive both therapies in either the AB or BA sequence, over two periods separated by a 10-week washout period.

**Table 1.** AB and BA sequence comparison.

Sequence	Sequence AB	Sequence BA
Frequency of treatment	50 minutes per day	50 minutes per day
Content of physiotherapy	Period 1: equine-assisted physiotherapy based on neuroproprioceptive “facilitation and inhibition” Period 2: standard individual outpatient physiotherapy	Period 1: standard individual outpatient physiotherapy Period 2: equine-assisted physiotherapy based on neuroproprioceptive “facilitation and inhibition”
Length of therapy	Period 1: 15 minutes twice a day Period 2: 30 minutes per day	Period 1: 30 minutes per day Period 2: 15 minutes twice a day
Length of therapeutic grooming	20 minutes per day	20 minutes per day
Environment	Period 2: treatment room Period 1: riding hall or outdoor terrain	Period 1: treatment room Period 2: riding hall or outdoor terrain
Involvement of specialists	Period 1: collaboration of physiotherapist with a team including a horse trainer and leader Period 2: physiotherapist only	Period 1: physiotherapist only Period 2: collaboration of physiotherapist with a team including a horse trainer and leader

The 6-day duration of each period was chosen to capture an immediate (short-term) response while avoiding undue fatigue in the children. In addition, the short, intensive therapeutic window allows for standardized application and limits the influence of concurrent rehabilitation. The long-term effect is captured 28 days after posttherapy as evidence of maintenance of effect and molecular adaptation.

The immediate effect of the 6-day intervention will be evaluated before and at the end of the program, focusing on functional performance (CHOP INTEND and MFM-20), muscle fatigue (sEMG), standardized postural and trunk control tests (TCMS, Modified Pediatric Reach Test, SCALE, and SATCo), respiratory function (spirometry), scoliotic progression (scoliometric measurements), and molecular biological changes (lncRNAs). 3D motion analysis will be performed using a 16-camera Qualisys Motion Capture System.

The long-term effect of the intervention (28 d after program completion) will be assessed to ensure clinical relevance and sustained benefit, focusing on quality of life (QoL) (standardized questionnaires), and psychomotor development (analyzed via home-video recordings).

**Figure 1.**
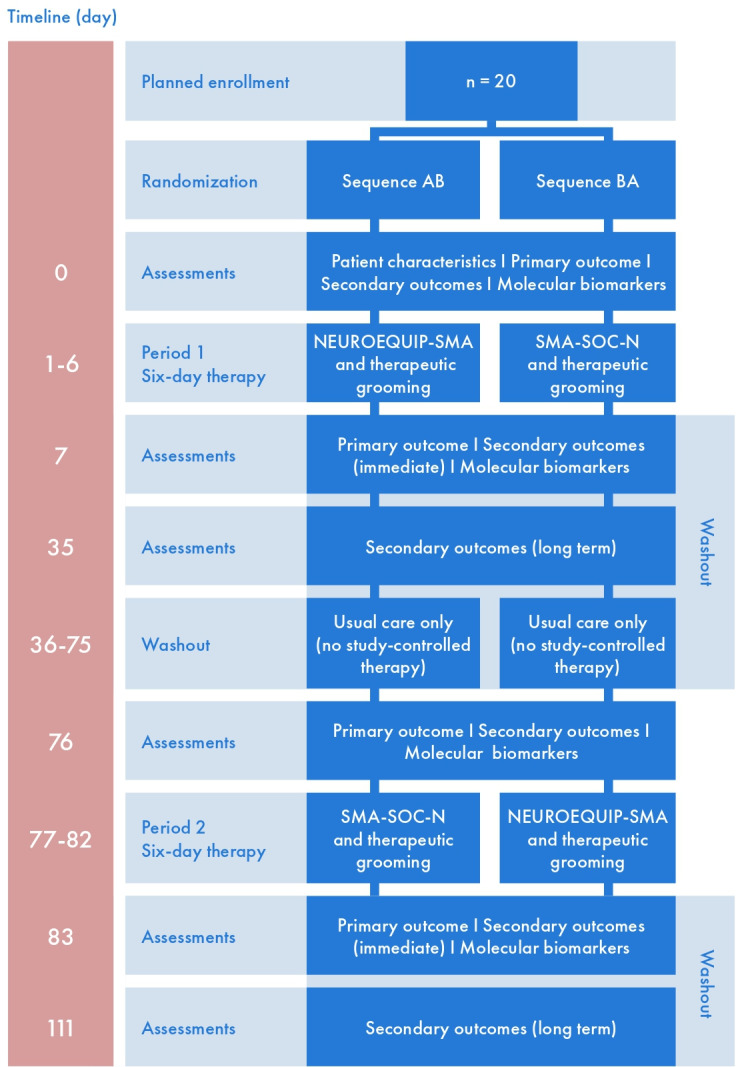
Study design.

The washout interval is a period during which participants do not receive any study-controlled therapy, only their usual care or ongoing therapeutic procedures independent of this study. The washout interval between Period 1 and Period 2 was set at 10 weeks. This duration was determined based on clinical experience and parental feedback, which suggested that short-term benefits of intensive physiotherapy can last approximately 6 weeks. The extended 4-week period beyond that allows sufficient time for residual therapeutic effects to dissipate while maintaining feasibility for participating families. After the completion of Period 2, another washout interval will follow (Figure 1 and Table 2).

**Table 2.** Schedule of assessments and interventions.

Timeline (day)		0	1‐6	7	8‐34	35	36‐75	76	77‐82	83	84‐110	111
	Before therapy	Pretherapy	Six-day therapy	Posttherapy		28 days posttherapy		Pretherapy	Six-day therapy	Posttherapy		28 days posttherapy
Enrollment	✓											
Eligibility screen	✓											
Informed consent	✓											
Allocation	✓											
Therapy												
Sequence AB			✓						✓			
Sequence BA			✓						✓			
Examination												
Patient characteristics		✓										
Primary outcome: CHOP INTEND^a^		✓		✓				✓		✓		
Secondary outcomes (immediate): MFM-20^b^, sEMG^c^, 3D-M^d^, SATCo^e^, TCMS^f^, mPRT^g^, SCALE^h^, SPM^i^, SCM^j^		✓		✓				✓		✓		
Secondary outcomes (long-term): ICF^k^, SDQ^l^, PedsQL^m^, and HVR^n^		✓				✓		✓				✓
Molecular biomarkers		✓		✓				✓		✓		
Washout period				✓	✓	✓	✓	✓		✓	✓	✓

a CHOP INTEND: Children's Hospital of Philadelphia Infant Test of Neuromuscular Disorders.

b MFM-20: Motor Function Measure-20.

c sEMG: surface electromyography.

d 3D-M: 3D motion.

e SATCo: Segmental Assessment of Trunk Control.

f TCMS: Trunk Control Measurement Scale.

g mPRT: Modified Pediatric Reach Test.

h SCALE: Selective Control Assessment of the Lower Extremity.

i SPM: spirometry measurement.

j SCM: scoliometer measurement.

k ICF: International Classification of Functioning, Disability, and Health.

l SDQ: Strengths and Difficulties Questionnaire.

m PedsQL: pediatric quality of life inventory.

n HVR: home video recording.

### Study Setting

Clinical examinations will be conducted at the College of Polytechnics Jihlava, which is equipped with advanced technology, including a 16-channel surface EMG system (DELSYS), spirometry, and Qualisys Motion Capture System with 16 cameras. The Mirákl Hippotherapy Center, a healthcare facility offering year-round Equine-Assisted Physiotherapy programs for children with special needs, has extensive experience and resources to support the study. LncRNA analysis will be performed at the Department of Medical Genetics, Third Faculty of Medicine, Charles University, which houses a state-of-the-art molecular biology laboratory specializing in genomic and biomarker research. The Institute of Computer Science at the Czech Academy of Sciences will contribute data management and statistical data analysis. Additionally, the Third Faculty of Medicine, Charles University, will provide institutional support for clinical research coordination, ensuring efficient execution and fostering interdisciplinary collaboration.

### Research and Management Team

The team includes coordinators, molecular biologists, specialists in 3D motion analysis and sEMG, physiotherapists, including those with expertise in Equine-Assisted Physiotherapy, horse trainers, and statisticians. The study coordinator will oversee recruitment, enrollment, eligibility assessment, and study conduct while providing participants with study details. All personnel involved in examinations will have no role in treatment allocation. A blinded assessor will perform clinical measurements, evaluate video recordings of clinical and functional tests, and collect questionnaire data. Molecular biologists will process peripheral blood samples for lncRNA analysis. Specialists will oversee 3D motion analysis, sEMG, and data processing. The statistician will manage dataset preparation, do statistical hypothesis testing, and interpret their results.

All therapists are trained to deliver interventions according to the protocol and working under the supervision of a senior therapist. Reproducibility is ensured by therapeutic manuals covering NEUROEQUIP-SMA, standard physiotherapy SMA-SOC-N, and therapeutic grooming with pre-defined parameter ranges. Outcome assessors undergo standardized training and calibration for clinical scales and clinical tests, spirometry, and scoliometer and instrumental measurements (eg, 3D motion capture and sEMG). Primary and secondary outcome video assessors are blinded to allocation and have ≥5 years of clinical experience with children with SMA.

### Sample Size

The study plans to include 20 children (10 per group), with each child receiving both therapies in the crossover design. When comparing the primary outcome (CHOP INTEND scores) between the NEUROEQUIP-SMA and standard therapies, a 2-sided paired *t* test with 80% power at a 5% significance level can detect an effect size of Cohen *d*=0.66, which is considered a moderate-to-large effect. The calculation of the detectable effect size for n=20, assuming normally distributed within-subject outcome differences, was performed using the pwr.t.test function from the R package *pwr* (version 1.3.0; R Foundation for Statistical Computing) [28].

### Recruitment

Participants will be simultaneously recruited from the database of the patient organization SMÁci, z. s., which registers 90% of children with SMA in the Czech Republic, while the remaining participants will be sourced from the database of the Mirákl Hippotherapy Center, o.p.s. All parents of children who meet the entry criteria will be contacted. Children will participate in 2 therapeutic cycles according to randomization, each time in a group of 10 children (the first 10 children enrolled will be assigned to the first therapeutic cycle). As of November 7, 2025, the patient organization has 215 registered members, including 45 children aged 2‐9.

### Eligibility Criteria

Participants will be eligible if they are between 2 and 9 years old and have a diagnosed SMA type I, II, or III with a stable health condition for at least 6 months, no other serious illnesses, and no intensive therapy 10 weeks before study intervention. Exclusion criteria will include hip luxation, allergies to horses or the stable environment, and an overwhelming fear of horses.

### Randomization and Blinding

The parents of children participating in the study will be fully informed. Upon giving written informed consent, their children will be randomly assigned to one of the treatment sequences. Block randomization with variable block sizes of 2 and 4 will be used to achieve balanced sequence sizes (AB and BA) within each therapeutic cycle of 20 children, due to organizational considerations. Allocation concealment will be maintained through opaque sealed envelopes. The randomization sequence will be generated using the online software Sealed Envelope [29]. Outcome assessors and investigators will remain blinded to minimize potential bias in outcome assessments. All examinations will take place in the same examination room under identical conditions, regardless of the assigned intervention. However, blinding will not be feasible for therapists, children with SMA, or their parents due to the nature of the interventions. Parents and children will not be told which of the therapies is considered the main intervention and which is the active comparator. To reduce bias, children and parents will be strongly advised not to disclose the allocated therapy during both the baseline and final assessments. While this is the planned procedure, it is acknowledged that practical factors, such as participant availability, medical status, or logistical constraints, may necessitate adjustments to the randomization or blinding processes to ensure the study’s feasibility and ethical conduct.

### Retention and Withdrawal and Participation Conditions

To support adherence, therapists will use their expertise and provide effective instruction to tailor each therapy session to the individual needs of the child, thereby enhancing adherence. Reasons for participant dropout will include discontinuing therapy for more than 2 missed physiotherapy sessions or significant noncooperation from the child. Participants will have the option to withdraw from the study at any time.

### Permitted and Prohibited Concomitant Care

During the 6-day intervention period, participants will not undergo any additional rehabilitation therapies. During the entire washout period, only standard outpatient physiotherapy will be allowed, with a maximum frequency of once per week.

### Intervention

**Figure 2.**
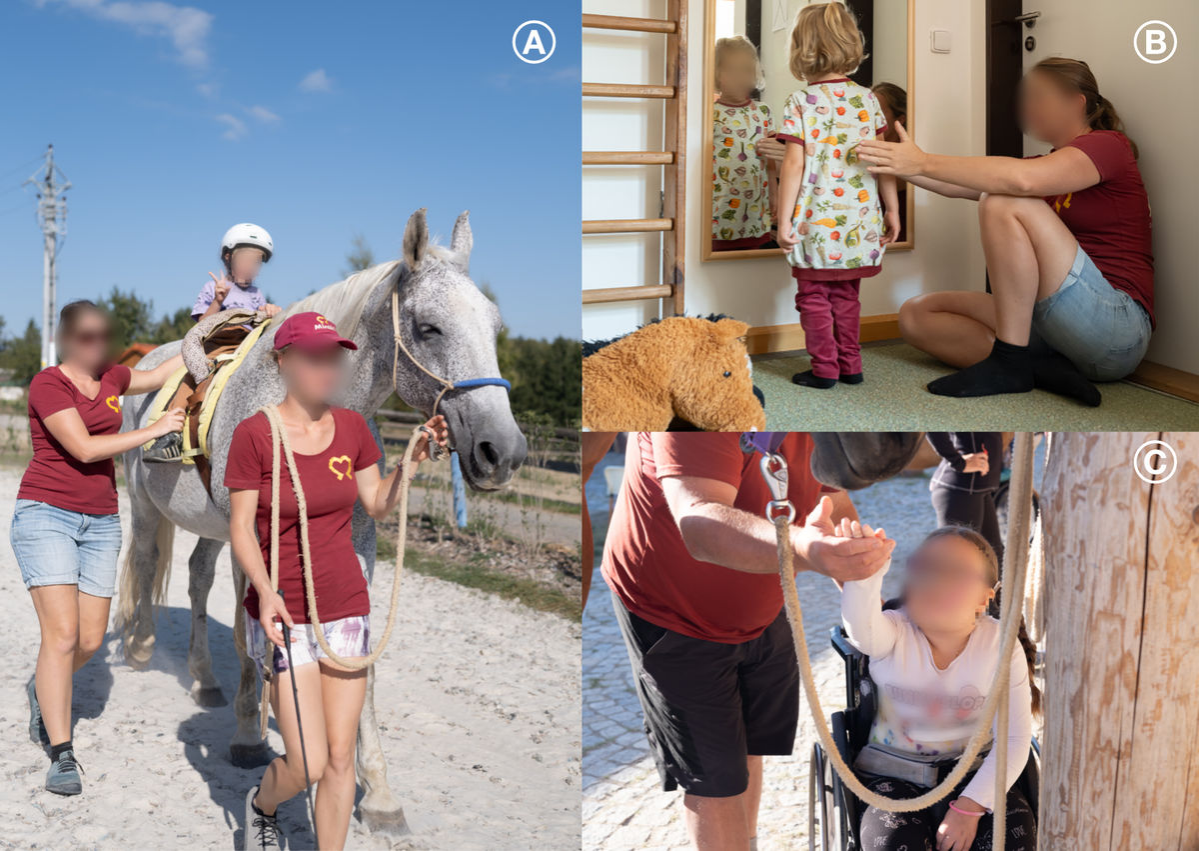
Photographs illustrating different therapeutic modalities.

The intervention group will undergo equine-assisted physiotherapy based on neuro-proprioceptive “facilitation and inhibition” (NEUROEQUIP-SMA; Figure 2A), while the active comparator group will receive standard individual outpatient physiotherapy based on neuroproprioceptive “facilitation and inhibition” (SMA-SOC-N; Figure 2B). Both groups will also receive therapeutic grooming (Figure 2C).

**Figure 3.**
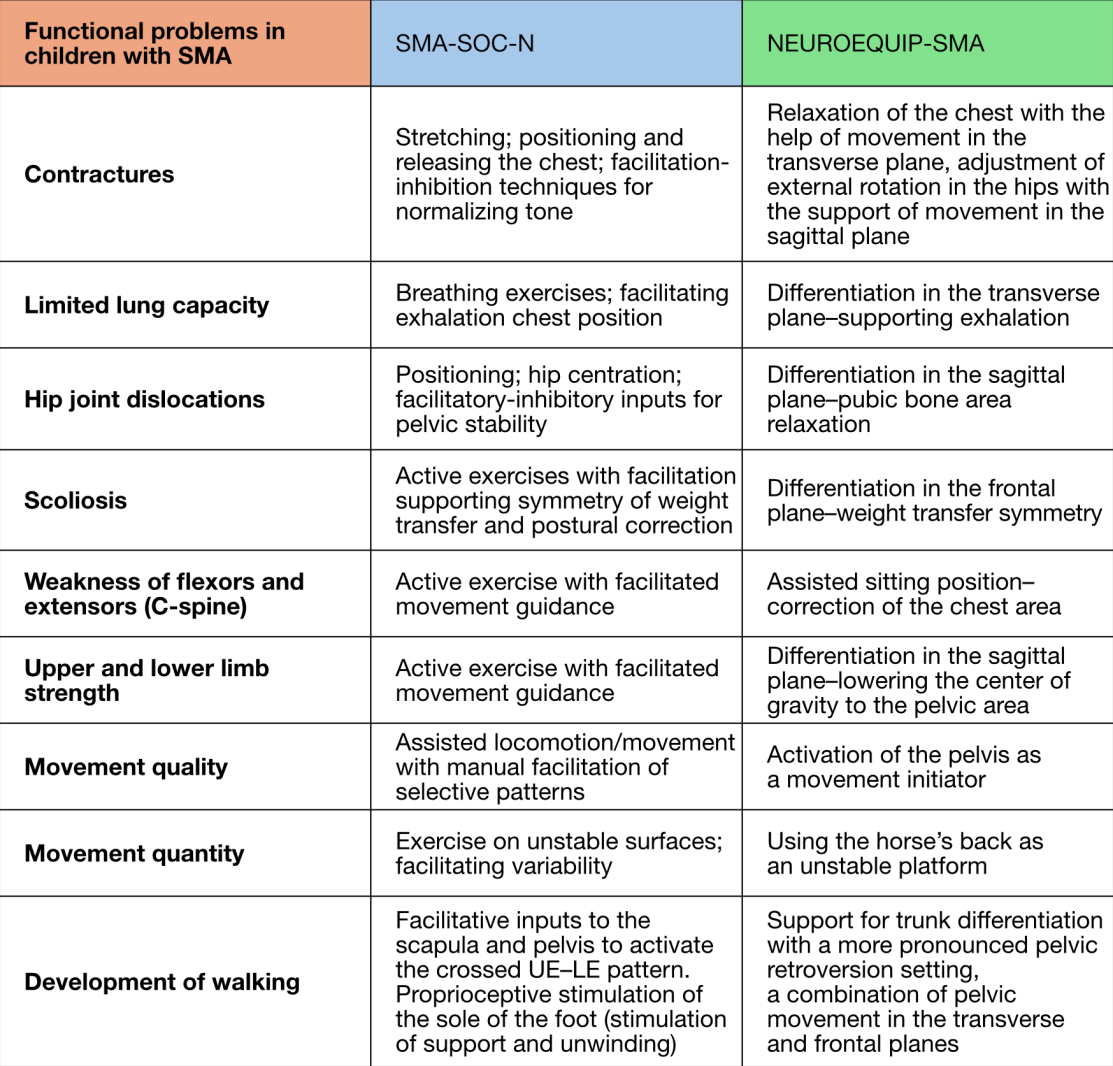
Comparison of functional problem management between intervention and active comparator groups. C-spine: cervical spine; UE: upper extremities; LE: lower extremities; SMA: spinal muscular atrophy.

Figure 3 shows how the intervention group differs from the active comparator group in addressing functional problems identified in both groups.

### Equine-Assisted Physiotherapy Based on Neuroproprioceptive “Facilitation and Inhibition”

Equine-Assisted Physiotherapy Based on Neuroproprioceptive “Facilitation and Inhibition” (NEUROEQUIP-SMA) differs from conventional hippotherapy in the way the horse’s movement stimulus is used and therapeutically processed. While conventional hippotherapy relies mainly on passive adaptation to the rhythmic movement of the horse, NEUROEQUIP-SMA is an individualized therapeutic method that activates proper motor function through the child’s consciously controlled response to the horse’s back movement. The goal is not to stabilize against the movement, but to accompany it, allowing natural postural reactions, primarily from the pelvic area. The child’s movement is secondary, naturally distributed, and rhythmically coordinated throughout the body. This method actively incorporates neuroproprioceptive “facilitation and inhibition,” using targeted afferent input, pelvic movement initiation, lowered center of gravity, and coordinated activation of muscle chains, which together enhance postural control and re-activate physiologic motor patterns.

**Figure 4.**
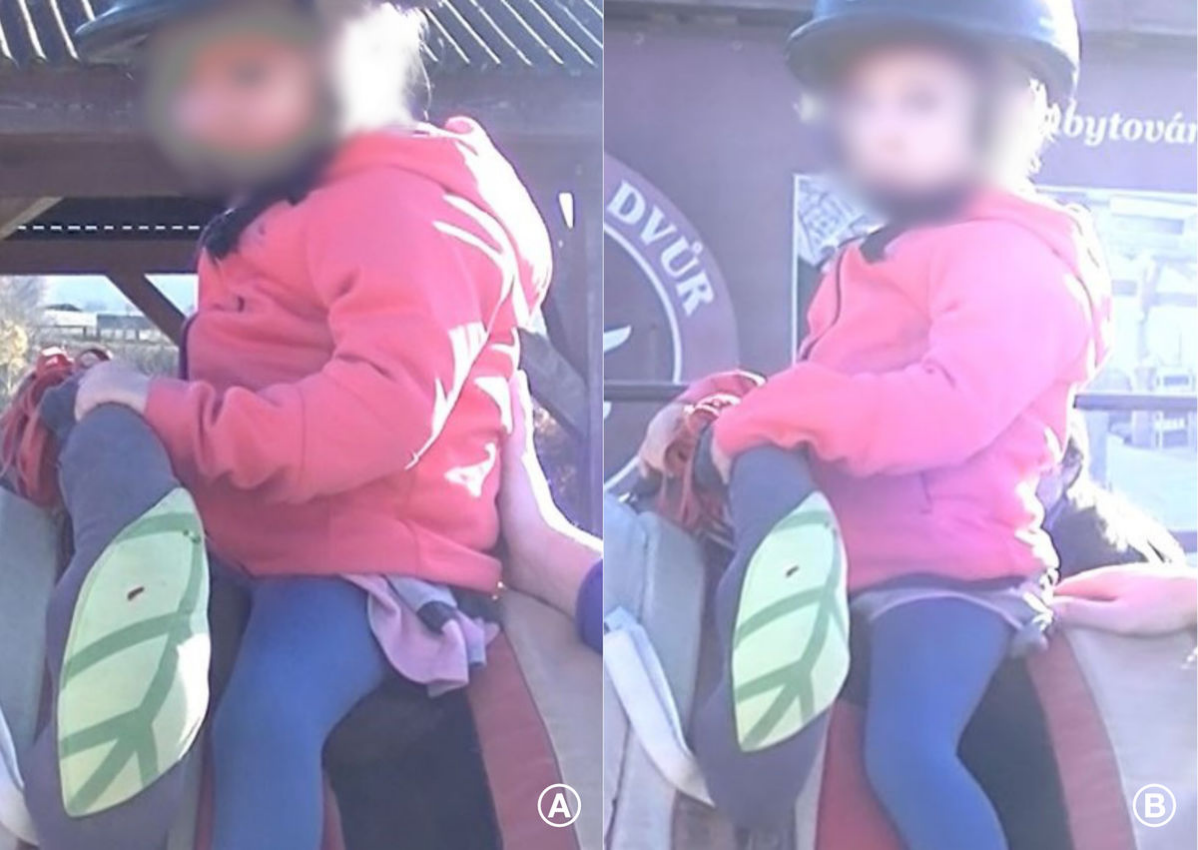
Example of a key therapeutic element: pelvic positioning correction. (A) Corrective position with correction by the therapist; the child is seated on the ischial tuberosity with external rotation of the hip joints. (B) The child presents with a pathological posture characterized by an anterior pelvic tilt and internal rotation of the hip joints.

The therapy, moreover, involves careful selection of the horse, therapeutic equipment, appropriate terrain, horse-leading technique, stride length, movement speed, horse head position, child’s position, use of positioning aids, and the physiotherapist’s manual contacts. The horse is chosen based on specific movement patterns that facilitate the desired motor responses. The pace, stride length, and direction of the horse’s movement are individually adapted to the child’s needs, with the therapist actively facilitating movement and correcting the child’s position using manual contacts or various types of positioning aids (Figure 4). The child is placed in various positions (sitting, lying, and assisted sitting) according to therapeutic goals and current capabilities.

The intervention is performed by a physiotherapist who is specially trained and certified in equine-assisted physiotherapy. Professional competence is obtained by completing a course accredited by the Ministry of Health of the Czech Republic [30,31] and working under professional supervision. The physiotherapist is part of a multidisciplinary team alongside a horse trainer and a horse leader. The horse must be specially trained for therapeutic purposes.

Therapy sessions are conducted either in a therapeutic riding hall or in an outdoor arena with equivalent surface conditions and a calm environment. The intervention is individually tailored based on the clinical assessment of the child, their current motor abilities, and therapeutic goals. Adjustments to meet therapy objectives may include changing the horse, modifying stride length, movement speed, horse-leading method, use of aids, child’s position, or the intensity of the therapist’s facilitation. Therapeutic tolerance is monitored through control or final examinations and feedback from parents. The success of the intervention is assessed based on the achievement of short-term therapy goals.

### Standard Individual Ambulatory Physiotherapy Based on Neuroproprioceptive “Facilitation and Inhibition” Principles (SMA-SOC-N)

Therapy follows the recommendations of the International Coordinating Committee for SMA Clinical Trials–Standard of Care, so it is individualized to address each child’s specific needs, taking into account their motor abilities and disease progression. The primary goals are to maintain or improve muscle strength, prevent contractures and deformities, support postural control, and optimize respiratory function. Therapeutic interventions include regular positioning, passive and active range of motion exercises, facilitation of functional movement patterns, and targeted training aimed at enhancing both stability and mobility [12].

The facilitation of functional movement patterns uses the principles of neuroproprioceptive “facilitation and inhibition,” which are commonly used in neurorehabilitation in the Czech Republic. It involves a combination of appropriate afferent stimuli designed to increase the excitability of motoneurons (lowering their excitability threshold) so that impulses from the central nervous system can be effectively transmitted, triggering neuronal discharge and muscle contraction. The goal is to maintain optimal conductivity and function of motor pathways, ensure appropriate excitability of control and regulatory structures, and adjust the activation level at the synapses of motor neurons in the anterior horns of the spinal cord as needed [32]. Their inclusion also allows for a better comparison of the effects between the active comparator and intervention groups, as both approaches use a similar degree of neuroproprioceptive “facilitation and inhibition” within motor patterns.

### Therapeutic Grooming

Therapeutic horse care in this protocol refers to a social activation service that takes place as structured, therapist-led work with a horse. Its goal is to support the child’s sensory and emotional regulation while safely and comfortably introducing them to the horse and stable environment. The intervention includes repeatable horse care activities (eg, brushing, mane combing, safe cleaning, and short horse leading), which are carried out according to our workplace’s internal standardized methodology. These activities allow children to engage naturally with the horse in a safe and supportive environment, promoting both physical and psychological development.

The therapy is guided by a trained therapist, who tailors the intervention to the individual needs of the child. This process involves selecting a suitable horse, adapting the environment, choosing appropriate aids, and applying specific touch techniques, all aimed at maximizing the therapeutic benefits. The goal extends beyond merely brushing the horse’s coat to stimulating sensory perception, regulating emotions and muscle tone, and enhancing body awareness.

Sessions take place in a calm and welcoming environment, where external noise and stressors are minimized, facilitating relaxation and allowing the child to focus on the present moment. The effectiveness of the intervention is evaluated through observations of the child’s interaction with the horse, emotional state before and after therapy, changes in motor skills, and long-term improvements in self-confidence, communication, and stress management.

Overall, therapeutic grooming offers a unique blend of physical care and emotional well-being. Through their connection with the horse, participants experience deep relaxation, inner balance, and the development of vital life skills.

Therapeutic cleaning is included in both groups to control the effect of interaction with the horse and staying in the stable or riding hall environment.

### Data Collection and Management

To support data consistency, instruments will be calibrated regularly. A statistician and coordinator will perform data checks. To ensure data collection, an Excel form will be created, and basic statistical reports will be generated regularly to check the completeness and validity of the data. Personal data will be pseudonymized using unique subject codes and stored on password-protected computers. Only the research team will have access to the identifiable data, and all records will be kept confidential in accordance with GDPR regulations. Anonymized data may be used for analysis and shared only as permitted by the informed consent.

### Outcome Measures

Patient characteristics will be collected, including age, gender, information about the disease and social anamnesis, last 5 results from the expanded Hammersmith Functional Motor Scale [33], date of last intensive hippotherapy (with the condition of more than 10 wk), the start and type of medication or rehabilitation, and use of compensatory aids prior to the program.

#### Primary Outcomes: CHOP INTEND

A clinical assessment tool used to evaluate motor function in infants with neuromuscular disorders measures their ability to perform specific movements and assesses the severity of motor impairments. Measurements are recorded during the test and are later evaluated by 2 independent physiotherapists (PP and MH) [34]. We will expand the standardized assessment with structured qualitative observations (compensatory strategies, movement initiation, body stabilization at the beginning of the movement, movement fluency, respiratory effort, etc). A higher score indicates a larger scope of motor skills.

#### Secondary Outcomes (Immediate)

##### 3D Motion-Based Evaluation of Postural Control, Breathing, and Coordination

###### Overview

**Figure 5.**
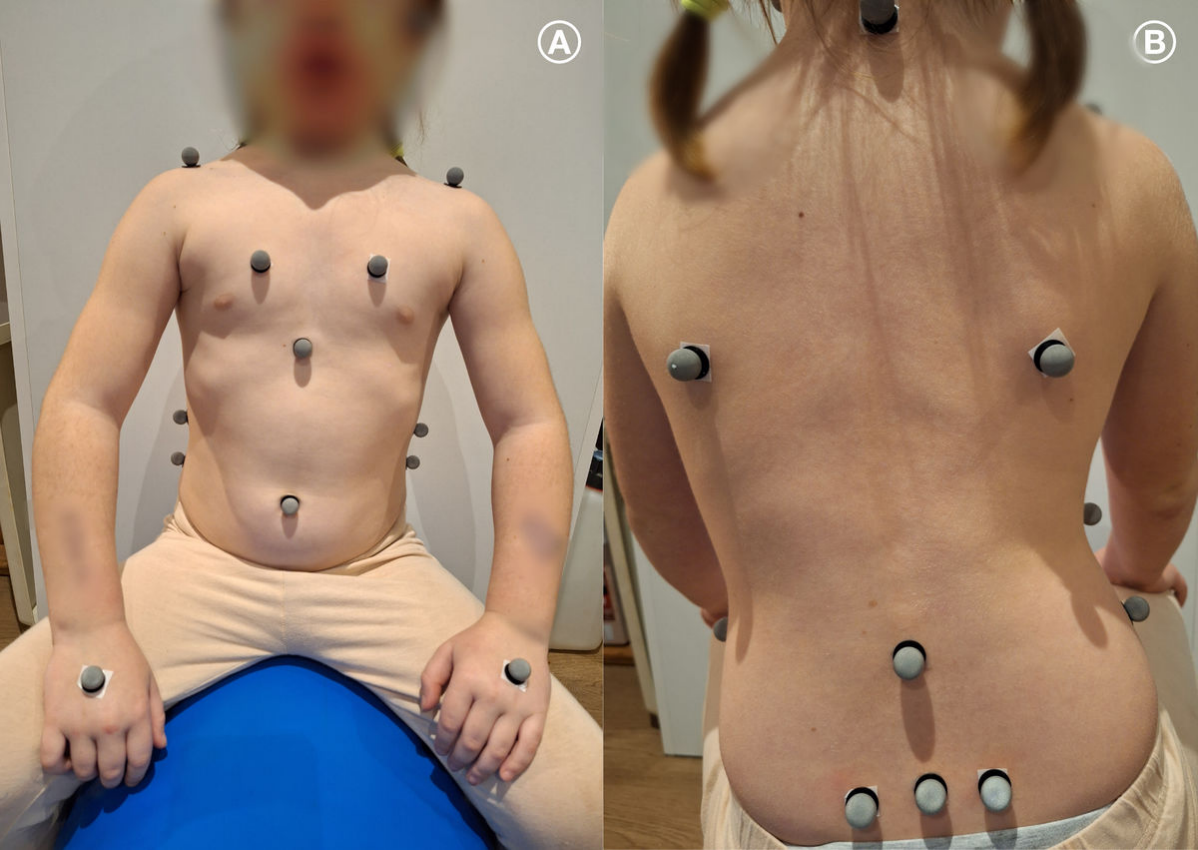
Locations of the 18 markers used for motion analysis.

All 3 tests share a common setup, in which the child sits straddling a cylinder with sensors attached. The cylinder is positioned within a calibrated 3D space and firmly secured to prevent movement. The child sits with feet on the ground and hips flexed to at least 90 degrees. In total, 18 markers are attached bilaterally at anatomically significant points, including the inferior angle of the scapula, sacroiliac joint, distal end of the clavicle, temporomandibular joint, dorsum of the hand, 4th rib (1-quarter proximal to the sternum), 10th rib (at the midpoint), top of the scapula, and hip. Additional markers are placed unilaterally at C7 (the thoracolumbar junction [Th/L]), L4 (the distal end of the sternum), and the umbilicus (Figure 5). Marker motion is captured using 16 Qualisys Motion Capture System cameras, allowing calculation of their spatial coordinates, from which position, velocity, and acceleration data are derived. Each measurement session lasts 30 seconds.

###### Test of Trunk and Cervical Spine Coordination Ability

**Figure 6.**
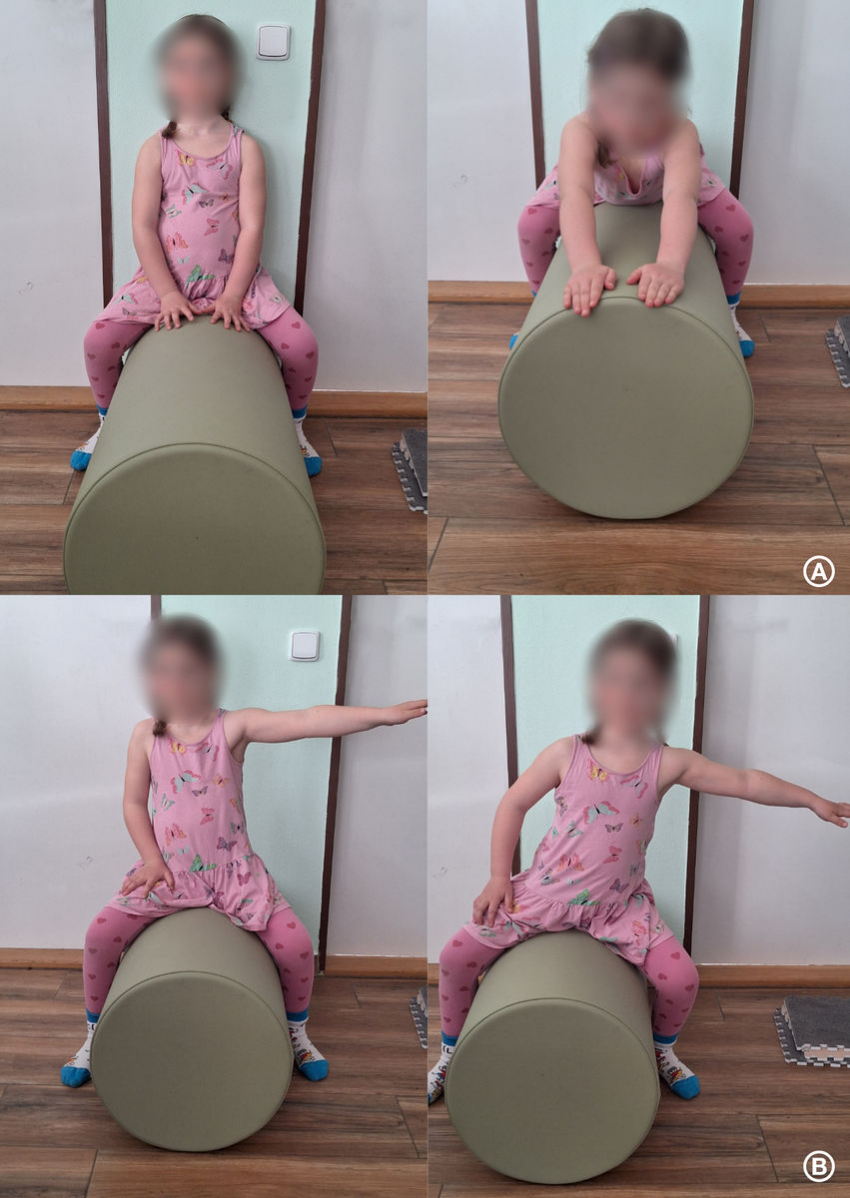
Movements of the upper limbs while sitting on the cylinder: (A) forward and backward and (B) reaching sideways.

The child performs repeated back-and-forth upper limb movements while sitting on the cylinder, typically 3‐6 repetitions. Key variables include the extent of thrust from the body’s center, the point at which cervical spine extension begins as compensation, and the coordination between markers located on the dorsum of the hand, C7 vertebra, and temporomandibular joint. A higher functional level is reflected by the child’s ability to synchronize cervical spine movements with trunk and upper limb actions over a longer period without compensatory cervical spine extensions (Figure 6).

###### Abdominal Breathing Ability Test

Over a 30-second period, the child is instructed to breathe deeply, aiming for 8‐12 respiratory cycles. Analysis focuses on the displacement of markers on the distal clavicles, distal sternum, L4, and the umbilicus during inspiration and expiration. The objective is to detect shifts toward an abdominal breathing pattern, with improved function indicated by increased distance between the Th/L and L4 markers relative to those around the umbilicus.

###### Ability to Work With the Center of Gravity

During the measurement, the child is asked to extend 1 upper limb sideways, and, depending on cooperation, may be encouraged to reach for a toy. Movements are performed 3 to 6 times within the 30-second recording period. This test evaluates stability and control over the center of gravity, with progress reflected in greater deviations of the markers from their initial positions after therapy (Figure 6).

### Muscle Fatigue

Muscle fatigue will be measured using sEMG. A 16-channel surface EMG system from DELSYS will be used to measure the activity of the m. obliquus externus abdominis for 30 seconds. The child will lie on their back, and the therapist will present a toy near the opposite knee to encourage the child to reach for it. The movement will be performed first in an isometric contraction, followed by a repetitive movement. We expect the child to be able to repeat the movement 5‐10 times during the 30-second measurement period. The EMG data will be processed using standard methods and normalized to the maximum EMG observed during isometric contraction to enable between-muscle comparisons (right and left side of the child). A lower median frequency and decreasing EMG amplitude during repeated movement indicate improved function, as the muscle performs the task more efficiently and with less effort. Additionally, a higher number of repetitions without significant changes in EMG characteristics reflects better function, suggesting functionally stronger or more coordinated muscle activation.

### SCALE

The SCALE test is a standardized clinical test used to assess selective voluntary motor control in the lower extremities. It assesses the ability to isolate joint movements during 5 tasks for each lower extremity. Each joint movement is scored from 0 to 2, and a higher total score indicates better selective motor control [35].

### The Modified Pediatric Reach Test

The Functional Reach Test is a simple, validated measure of dynamic sitting or standing balance that evaluates the maximal distance an individual can reach forward beyond arm’s length while maintaining a fixed base of support. It is a reliable indicator of trunk stability and balance control in children with various neuromuscular conditions [36]. Longer reach distances indicate better dynamic postural control and balance performance [37].

### Spirometry Measurement

Spirometry will be performed according to the international standards of the European Respiratory Society [38]. The patient will be fitted with a nose clip, then they will breathe into the spirometer through the mouthpiece while sitting on the cylinder. Each child receives the same instructions: Take a deep breath and then exhale as quickly and forcefully as possible. The test will be repeated 3 times in a row. It will be performed using the Vitalograph Asma-1 device. Forced expiratory volume in 1 second and peak expiratory flow will be measured [39]. Higher values indicate better function.

### Scoliometer Measurement

The measurement will be performed using a scoliometer according to the standardized procedure for assessing the angle of trunk rotation (ATR). The scoliometer is a noninvasive diagnostic tool based on the principle of a spirit level, which allows for the orientation detection of trunk asymmetry and possible rotation of the vertebrae. The child being examined will sit on a cylinder with the upper limbs relaxed along the body. The child will then be asked to bend forward as much as possible while sitting. The scoliometer is placed on the spine so that the center of the device exactly copies the spinous processes. The measurement will be performed at 2 levels: Th/L transition (thoracolumbar transition) and Level 2/3 of the thoracic spine. At each level, the ATR value will be recorded as well as the side to which the trunk rotation is directed. Each measurement will be performed 3 times in succession, and the average of the values will be used. A higher ATR value indicates greater axial rotation of the trunk and thus a higher degree of possible scoliotic curvature [40].

### MFM-20

The MFM-20 is a validated quantitative scale assessing motor skills in children with neuromuscular disorders aged 2‐7 years. It contains 20 items covering standing, transfer, axial and proximal motor functions, and distal motor control. The total score is expressed as a percentage of the maximum possible score, with higher scores indicating better motor performance. The MFM-20 has been validated in SMA and Duchenne muscular dystrophy and is sensitive to changes over time [33].

### SATCo

The SATCo is a standardized test designed to identify the highest level of segmental trunk control in sitting. It assesses static, active, and reactive control at 7 levels of trunk support. Although originally developed for children with cerebral palsy, it has been used in other pediatric neuromotor populations to evaluate trunk stability and postural development [41]. Higher scores represent a greater degree of segmental trunk control [42].

### TCMS

The TCMS is a clinical assessment tool that evaluates trunk control during static and dynamic sitting balance tasks. It measures selective movement control in the trunk and coordination between upper and lower body segments. Although validated primarily in children with cerebral palsy, it is also used to assess postural control in children with other neuromotor disorders such as SMA [43]. The total score ranges from 0 to 58, with higher scores indicating better trunk control [44].

### Secondary Outcomes (Long-Term)

#### Quality of Life Questionnaire

Parents of children with SMA will complete a special questionnaire. The ICF score set, specifically the ICF-based Documentation Form for the category “Children with Cerebral Palsy Brief” (for children under 6 y old), will be used to evaluate the QoL [45]. Although the ICF-based Documentation Form was originally developed for children with cerebral palsy, it was selected in this trial due to its better applicability in clinical practice and its ability to capture functional and quality-of-life domains relevant also to children with SMA.

#### The Pediatric Quality of Life Inventory Generic Core Scales

Parents of children with SMA will also complete the Pediatric Quality of Life Inventory Generic Core Scales - Parent Proxy Report. The assessment tool, designed to evaluate health-related QoL in children, consists of 23 items across 4 domains (physical, emotional, social, and school functioning). It is used to monitor longitudinal changes in perceived QoL and to evaluate the effectiveness of clinical and rehabilitative interventions. Higher scores indicate better QoL [46].

#### Strengths and Difficulties Questionnaire

The Strengths and Difficulties Questionnaire is a widely used behavioral screening tool assessing psychological attributes across 5 domains: emotional symptoms, conduct problems, hyperactivity or inattention, peer relationship problems, and prosocial behavior. It is suitable for children aged 2 to 17 years and provides a total difficulties score reflecting overall psychosocial well-being. Although originally validated in the general pediatric population, it has been successfully applied in children with chronic or neuromuscular disorders to assess emotional and social adaptation related to health status. Higher total scores indicate greater difficulties, while higher prosocial subscale scores indicate better social functioning [47].

#### Home Video Recording

Parents will send home video recordings of their child’s movement behavior. The video recordings will be evaluated by 2 independent physiotherapists who will assess quality and extent of the children’s movement. Movement of greater quality or scope indicates better function.

### Molecular Biomarkers

For lncRNA analysis, peripheral blood will be collected in the evening before and after the completion of the rehabilitation course, directly at the accommodation site for families with children with SMA. Given the collection site, blood samples will be immediately frozen using dry ice and transported to the laboratory, where they will be stored at -80°C. Rapid freezing eliminates RNA degradation as much as possible. RNA will be isolated from gently thawed samples [48] using the NucleoSpin RNA blood kit (Macherey-Nagel), which is specially designed for RNA isolation from frozen blood. The expected average yield is 7 µg per 1 ml of blood. Total RNA will be transcribed into cDNA using reverse transcriptase. The expression of selected lncRNAs will be detected by qPCR using hydrolysis probes designed specifically for the selected lncRNAs. Relative quantification with selected reference genes will be used for expression analysis. Reference genes will be selected during qPCR protocol optimization using the R program and recommended packages (eg, NormFinder). All qPCR experiments will be done in accordance with MIQE (Minimum Information for Publication of Quantitative Real-Time PCR Experiments) guidelines.

### Statistical Analysis and Software

Data will be presented as means (SDs), medians (IQRs), or as absolute numbers with percentages, as appropriate. The quantitative outcomes will be expressed as differences relative to the corresponding pretherapy values (post – pre), while qualitative outcomes will be categorized as improved, unchanged, or worsened compared to pretherapy. For each quantitative outcome, the treatment effect will be evaluated by testing within-subject differences between NEUROEQUIP-SMA therapy (A) and SMA-SOC-N therapy (B) using a paired *t* test. Although no period effect (eg, due to disease progression or season) is expected, it will be assessed by comparing within-subject differences (Period 1 vs Period 2) using a paired *t* test. The study was designed to avoid carryover effects through a long washout period; nonetheless, carryover effects will be explored by comparing between-subject differences in outcome sums (Sequence AB vs Sequence BA) using a 2-sample *t* test. If the normality assumption is not met, the exact Wilcoxon signed-rank test will be used instead of the paired *t* test, and the exact Wilcoxon rank-sum test instead of the 2-sample *t* test. Normality will be assessed both graphically (eg, Q–Q plot) and using the Shapiro–Wilk test. Similarly, for qualitative outcomes, the effects of treatment, period, and carryover will be assessed using the McNemar test for within-subject differences and Fisher exact test for between-subject differences.

When conducting multiple comparisons, the achieved levels of significance (*P* values) will be adjusted using a correction method, such as the Holm-Bonferroni method. All tests will be 2-sided, with *P* values less than .05 considered statistically significant. Effect sizes will be reported alongside *P* values to provide insight into the magnitude of observed changes. Sensitivity analyzes for outliers will be included, comparing results with and without these subjects. Data analysis will be performed using R software (R Development Core Team) [49]. Missing data will not be imputed due to the small sample size, which could make imputation unstable; instead, an available-case analysis will be performed, and the pattern of missing data will be explored. No interim analyzes are planned. The trial will be conducted in full as described, unless terminated early for ethical or safety reasons upon recommendation of the principal investigator or the ethics committee.

### Harms

The physiotherapists are qualified professionals who actively approach all subjects with respect to their individual impairment. The horses performing the therapy in the study have specialized examinations, and the trainers are professionals with many years of experience to sufficiently ensure safety during NEUROEQUIP-SMA and therapeutic care. The program is individualized to avoid burdening or harming the patient. All adverse events (AEs) must be reported to the principal investigator and the appropriate specialist. All AEs will be monitored and systematically recorded by the treating physiotherapists during the study using a predefined reporting form. AEs will be classified according to severity (mild, moderate, severe) and potential relationship to the intervention (unrelated, possibly related, and definitely related) and will be reviewed by the principal investigator and a designated clinical specialist. Serious AEs will be reported immediately to the ethics committee. In the event of any injury related to the intervention, appropriate clinical care will be provided, but no financial compensation will be provided.

### Ethical Considerations

The multicentric Ethics Committee of the Third Faculty of Medicine of Charles University has approved the study under the code UK3LF/658559/12025, based on submitted informed consent forms. The results of the study will be disseminated to the public and the scientific and medical community (published in journal articles and conferences). Anonymized data and outcome data may be published for research purposes (for meta-analysis, systematic reviews, etc) on request. This study does not include a Data Monitoring Committee, as the intervention is short-lasting, low risk, and nonpharmacological in nature. Oversight is ensured by the principal investigator and the ethics committee. Any modifications to the study design will be submitted to the ethics committee for review, and the informed consent form will be revised accordingly. No monetary compensation was provided to participants. Written informed consent for publication of identifiable images was obtained from the legal guardians of all participating children.

## Results

All potentially eligible study participants will be contacted in December 2025. Data collection will take place from February 2026 to May 2026. The start of statistical analyzes is planned for September 2026. In the crossover design, 20 children are planned to receive both therapies over 2 periods, enabling within-subject comparisons. The washout period of sufficient length will minimize potential carryover effect between periods. A paired *t* test comparing the primary outcome between NEUROEQUIP-SMA and SMA-SOC-N therapies in 20 children will have 80% power to detect moderate-to-large effect size (Cohen *d*=0.66) at a 5% significance level. Analyses of other outcomes are exploratory in nature and aimed at informing future hypothesis-driven research.

Although period and carryover effects on the outcome are not expected, they will also be assessed alongside the treatment effects; however, all these effects will be analyzed separately due to the small sample size. Along with *P* values, effect sizes will be reported to reflect the magnitude of changes. We would like to publish the collected data at the beginning of 2027.

## Discussion

### Anticipated Findings

This protocol presents the design of a clinical trial comparing an innovative NEUROEQUIP-SMA approach with standard physiotherapy in children with SMA. This is the first study of its kind to evaluate the effect of EAT based on the principles of neuroproprioceptive “facilitation and inhibition.” We plan to support the results with molecular data from lncRNA analysis. Based on previous studies [25], we predict that the expression levels of selected lncRNAs (GAS5, H19, MALAT1, MEG3, NEAT1, and PARTICLE) will change as a result of therapy. These lncRNAs have not been sufficiently studied in relation to SMA, but based on their mechanism of action and detection in other diseases, we postulate their deregulation in the blood of patients with SMA. This study may be the first step toward understanding the involvement of lncRNAs in the pathophysiology of this disease and their use as prognostic markers [48]. The uniqueness of the study also lies in the specific target group: children who have undergone gene therapy. We anticipate that intensive EAT based on the principles of neuroproprioceptive “facilitation and inhibition” will be proven and well tolerated in children with SMA. We also expect that the protocol will allow for the capture of changes at the molecular, biomechanical, and clinical levels.

In the Czech Republic, the Nusinersen substitution therapy has been included in the system of reimbursed care as regular reimbursement since 2018 [50], while the Zolgensma gene therapy was approved by the Ministry of Health of the Czech Republic in April 2020 before registration as preauthorized use [51]. Physiotherapists are thus in current practice dealing with diverse groups of children: those treated with substitution therapy for a long time, those treated with a combination of both approaches, and those who have received gene therapy very early. We have a new generation of children who can be expected to slow or stop the progression of the disease [52,53].

Based on these changes, there is a need to revise existing rehabilitation strategies in clinical practice. Some physiotherapists are recommending an increase in the frequency and intensity of therapy [54] and new standards of care are needed to better reflect patient expectations and variable motor skills [55]. Although pharmacological treatment significantly improves survival, motor function often remains limited and is accompanied by pathological compensatory mechanisms [12,56,57]. Targeted correction of these functions with appropriate rehabilitation approaches is therefore essential. For this reason, an intensive six-day course and the therapeutic neuroproprioceptive principle were chosen.

### Comparison With Prior Work

There is currently only 1 study examining the effect of EAT in children with SMA. Lemke et al [23] focused on subjective assessment of the effects of EAT using a questionnaire survey. In contrast, our study evaluates the direct response to a therapeutic intervention, which we are able to observe at the clinical, biomechanical, and molecular levels, including comparison with a standardized SMA-SoC procedure.

Research on conventional rehabilitation approaches for children with SMA has so far focused mainly on maintaining existing function, preventing contractures, and promoting self-sufficiency [6,12,58]. Most of these were designed before the onset of gene therapy, when disease progression was considered inevitable and often lacked targeted monitoring of compensatory mechanisms. In our study, we augmented standardized tests with qualitative assessments to capture potential re-education of pathological patterns. In the era of genetic treatments, we consider the monitoring of movement quality to be essential, and the existing literature has not yet addressed this milestone.

Cammarano et al [59] summarize that rehabilitation care for SMA, whether before or after genetic treatment, lacks standardization, high-quality randomized trials, and comprehensive multidomain evaluations. Our study addresses this requirement: it includes children treated with both replacement and genetic therapy.

### Limitations of This Study

As the participants are children, the results are partly affected by their level of cooperation and willingness to participate. Although we carefully selected the assessment tools to minimize this source of variability, some degree of bias cannot be excluded. Due to the extensive test battery and possible fatigue effects, we administer each test only once the child is able to respond appropriately. Limitations can also be seen in the selection of the quality-of-life test. Although the ICF tool is practical and most commonly used, it was originally validated for cerebral palsy, which may reduce its specificity for SMA.

The functional focus, specificity, precision, and technique inherent in the NEUROEQUIP-SMA methodology are key to the overall therapeutic effect and can account for minor differences in results. Since SMA is a rare genetic disease, participants were recruited from across the country. The number of participants is limited by the rarity of the disease, which limits the statistical power and generalizability of the results. Bias may also occur due to the established randomization process, where therapists and families could not be blinded. Due to travel restrictions, the long-term effects of the NEUROEQUIP-SMA intervention can only be partially assessed. Furthermore, given the severity of the disease, it is likely that children will engage in further physiotherapy interventions approximately 4 weeks after completing our treatment program. Although the small sample size limits the use of more complex statistical models, such as linear mixed models, the results will provide a solid basis for future research.

Given the crossover design, potential attrition before the second half of the study is considered a methodological risk. To mitigate this risk, families will be engaged through regular communication, stable scheduling, and motivation supported by the same therapeutic team across both parts of the study.

### Conclusions

The proposed evaluation methods provide a comprehensive assessment of the NEUROEQUIP-SMA approach and can serve as a foundation for future comparisons of different methodologies in equine-assisted therapy. The findings of this study have the potential to inform and shape rehabilitation strategies for children with SMA receiving genetic or substitution pharmacotherapy. The results will be shared with the professional and lay public and translated into clinical practice. We hope that this protocol will spark a discussion about possible changes in intervention for children with SMA on genetic therapy and highlight the need for larger and longer-term multicenter studies to verify durability of effect, clinical efficacy, and transferability to practice.

## Supplementary material

10.2196/83266 Checklist 1 CONSORT-EHEALTH checklist (V1.6.1).

10.2196/83266 Checklist 2 SPIRIT checklist.

## Abbreviations

AE: adverse event
CHOP INTEND: Children's Hospital of Philadelphia Infant Test of Neuromuscular Disorders
ICF: International Classification of Functioning, Disability, and Health
lncRNA: long noncoding RNA
MFM-20: Motor Function Measure-20
QoL: quality of life
SCALE: Selective Control Assessment of the Lower Extremity
sEMG: surface electromyography
SMA: spinal muscular atrophy
SPIRIT: Standard Protocol Items: Recommendations for Interventional Trials
