# Supplementary material for: Immediate and Sustained Effects of Intensive Equine-Assisted Physiotherapy Based on Neuroproprioceptive “Facilitation and Inhibition” on Psychomotor Development, Clinical Functions, Quality of Life, and Molecular Biological Indicators in Children With Spinal Muscular Atrophy: Protocol for a Crossover Randomized Controlled Trial
Source: JMIR Res Protoc. 2026 Feb 19;15:e83266. doi: 10.2196/83266 (PMC12919904; doi:10.2196/83266)
Supplement: Checklist 1 [file resprot-v15-e83266-s001.pdf]

CONSORT-EHEALTH (V 1.6.1) - Submission/Publication Form

The CONSORT-EHEALTH checklist is intended for authors of randomized trials evaluating web-based and Internet-based applications/interventions, including mobile interventions, electronic games (incl multiplayer games), social media, certain telehealth applications, and other interactive and/or networked electronic applications. Some of the items (e.g. all subitems under item 5 - description of the intervention) may also be applicable for other study designs.

The goal of the CONSORT EHEALTH checklist and guideline is to be
a) a guide for reporting for authors of RCTs,
b) to form a basis for appraisal of an ehealth trial (in terms of validity)

CONSORT-EHEALTH items/subitems are MANDATORY reporting items for studies published in the Journal of Medical Internet Research and other journals / scientific societies endorsing the checklist.

Items numbered 1., 2., 3., 4a., 4b etc are original CONSORT or CONSORT-NPT (non-pharmacologic treatment) items.
Items with Roman numerals (i., ii, iii, iv etc.) are CONSORT-EHEALTH extensions/clarifications.

As the CONSORT-EHEALTH checklist is still considered in a formative stage, we would ask that you also RATE ON A SCALE OF 1-5 how important/useful you feel each item is FOR THE PURPOSE OF THE CHECKLIST and reporting guideline (optional).

Mandatory reporting items are marked with a red *.
In the textboxes, either copy & paste the relevant sections from your manuscript into this form - please include any quotes from your manuscript in QUOTATION MARKS,
or answer directly by providing additional information not in the manuscript, or elaborating on why the item was not relevant for this study.

YOUR ANSWERS WILL BE PUBLISHED AS A SUPPLEMENTARY FILE TO YOUR PUBLICATION IN JMIR AND ARE CONSIDERED PART OF YOUR PUBLICATION (IF ACCEPTED).
Please fill in these questions diligently. Information will not be copyedited, so please use proper spelling and grammar, use correct capitalization, and avoid abbreviations.

DO NOT FORGET TO SAVE AS PDF _AND_ CLICK THE SUBMIT BUTTON SO YOUR ANSWERS ARE IN OUR DATABASE !!!

Citation Suggestion (if you append the pdf as Appendix we suggest to cite this paper in the caption):
Eysenbach G, CONSORT-EHEALTH Group
CONSORT-EHEALTH: Improving and Standardizing Evaluation Reports of Web-based and Mobile Health Interventions
J Med Internet Res 2011;13(4):e126

URL: http://www.jmir.org/2011/4/e126/
doi: 10.2196/jmir.1923
PMID: 22209829

capkova.ka1@gmail.com Přepnout účet

Není sdíleno

Koncept uložen

* Označuje povinnou otázku

Your name *

First Last

Kateřina

Primary Affiliation (short), City, Country *

University of Toronto, Toronto, Canada

Charles University, Prague, Czechia

Your e-mail address *

abc@gmail.com

katerina.marikova@chmirakl.cz

Title of your manuscript *

Provide the (draft) title of your manuscript.

Immediate and Sustained Effects of Intensive Equine-Assisted Physiotherapy Based on Neuroproprioceptive “Facilitation and Inhibition” on Psychomotor Development, Clinical Functions, Quality of Life, and Molecular Biological Indicators in Children with Spinal Muscular Atrophy: Protocol for a Randomized Crossover-Controlled Trial

Name of your App/Software/Intervention *

If there is a short and a long/alternate name, write the short name first and add the long name in brackets.

NEUROEQIP-SMA (Neuroproprioceptive Equine

Evaluated Version (if any)

e.g. "V1", "Release 2017-03-01", "Version 2.0.27913"

Protocol version 2.0, 14 November 2025

Language(s) *

What language is the intervention/app in? If multiple languages are available, separate by comma (e.g. "English, French")

English

URL of your Intervention Website or App

e.g. a direct link to the mobile app on app in appstore (itunes, Google Play), or URL of the website. If the intervention is a DVD or hardware, you can also link to an Amazon page.

www.chmirakl.cz

URL of an image/screenshot (optional)

N/A – intervention is not digital; no screenshot exists.

Accessibility *

Can an enduser access the intervention presently?

- ○ access is free and open
- ○ access only for special usergroups, not open
- ○ access is open to everyone, but requires payment/subscription/in-app purchases
- ○ app/intervention no longer accessible
- ◉ Jiné: The intervention is an in-person physiotherapy program provided only v

Primary Medical Indication/Disease/Condition *

e.g. "Stress", "Diabetes", or define the target group in brackets after the condition, e.g. "Autism (Parents of children with)", "Alzheimers (Informal Caregivers of)"

Spinal Muscular Atrophy (pediatric population)

Primary Outcomes measured in trial *

comma-separated list of primary outcomes reported in the trial

3D Motion-Based Evaluation of Postural Contr

Secondary/other outcomes

Are there any other outcomes the intervention is expected to affect?

Pediatric Quality of Life Inventory™ (PedsQL™ 4.0), Strengths and Difficulties Questionnaire (SDQ), home video recordings of movement behaviour, peripheral blood sampling for analysis of selected long non-coding RNAs (IncRNAs: GAS5, H19, MALAT1, MEG3, NEAT1, PARTICLE)

Recommended "Dose" *

What do the instructions for users say on how often the app should be used?

- ○ Approximately Daily
- ○ Approximately Weekly
- ○ Approximately Monthly
- ○ Approximately Yearly
- ○ "as needed"
- ◉ Jiné: The intervention is delivered as an intensive 6-day in-person physiother

Approx. Percentage of Users (starters) still using the app as recommended after 3 months *

- ◉ unknown / not evaluated
- ○ 0-10%
- ○ 11-20%
- ○ 21-30%
- ○ 31-40%
- ○ 41-50%
- ○ 51-60%
- ○ 61-70%
- ○ 71%-80%
- ○ 81-90%
- ○ 91-100%
- ○ Jiné:

Overall, was the app/intervention effective? *

- ( ) yes: all primary outcomes were significantly better in intervention group vs control
- ( ) partly: SOME primary outcomes were significantly better in intervention group vs control
- ( ) no statistically significant difference between control and intervention
- ( ) potentially harmful: control was significantly better than intervention in one or more outcomes
- (•) inconclusive: more research is needed
- ( ) Jiné:

Article Preparation Status/Stage *

At which stage in your article preparation are you currently (at the time you fill in this form)

- ( ) not submitted yet - in early draft status
- ( ) not submitted yet - in late draft status, just before submission
- ( ) submitted to a journal but not reviewed yet
- (•) submitted to a journal and after receiving initial reviewer comments
- ( ) submitted to a journal and accepted, but not published yet
- ( ) published
- ( ) Jiné:

Journal *

If you already know where you will submit this paper (or if it is already submitted), please provide the journal name (if it is not JMIR, provide the journal name under "other")

- ○ not submitted yet / unclear where I will submit this
- ○ Journal of Medical Internet Research (JMIR)
- ○ JMIR mHealth and UHealth
- ○ JMIR Serious Games
- ○ JMIR Mental Health
- ○ JMIR Public Health
- ○ JMIR Formative Research
- ○ Other JMIR sister journal
- ◉ Jiné: JMIR Research Protocols

Is this a full powered effectiveness trial or a pilot/feasibility trial? *

- ◉ Pilot/feasibility
- ○ Fully powered

Manuscript tracking number *

If this is a JMIR submission, please provide the manuscript tracking number under "other" (The ms tracking number can be found in the submission acknowledgement email, or when you login as author in JMIR. If the paper is already published in JMIR, then the ms tracking number is the four-digit number at the end of the DOI, to be found at the bottom of each published article in JMIR)

- ○ no ms number (yet) / not (yet) submitted to / published in JMIR
- ◉ Jiné: #83266

TITLE AND ABSTRACT

1a) TITLE: Identification as a randomized trial in the title

1a) Does your paper address CONSORT item 1a? *

I.e does the title contain the phrase "Randomized Controlled Trial"? (if not, explain the reason under "other")

- (selected) yes
- ( ) Jiné:

1a-i) Identify the mode of delivery in the title

Identify the mode of delivery. Preferably use “web-based” and/or “mobile” and/or “electronic game” in the title. Avoid ambiguous terms like “online”, “virtual”, “interactive”. Use “Internet-based” only if Intervention includes non-web-based Internet components (e.g. email), use “computer-based” or “electronic” only if offline products are used. Use “virtual” only in the context of “virtual reality” (3-D worlds). Use “online” only in the context of “online support groups”. Complement or substitute product names with broader terms for the class of products (such as “mobile” or “smart phone” instead of “iphone”), especially if the application runs on different platforms.

	1	2	3	4	5	
subitem not at all important	( )	(selected)	( )	( )	( )	essential

Zrušit výběr

Does your paper address subitem 1a-i? *

Copy and paste relevant sections from manuscript title (include quotes in quotation marks "like this" to indicate direct quotes from your manuscript), or elaborate on this item by providing additional information not in the ms, or briefly explain why the item is not applicable/relevant for your study

This item is not applicable.
The intervention is not web-based, mobile, electronic, or digitally delivered; it is an in-person equine-assisted physiotherapy program. Therefore, including terms such as “web-based,” “mobile,” “electronic,” or similar modality descriptors in the title would be misleading and not relevant.

1a-ii) Non-web-based components or important co-interventions in title

Mention non-web-based components or important co-interventions in title, if any (e.g., “with telephone support”).

	1	2	3	4	5	
subitem not at all important	(●)	○	○	○	○	essential

Zrušit výběr

Does your paper address subitem 1a-ii?

Copy and paste relevant sections from manuscript title (include quotes in quotation marks "like this" to indicate direct quotes from your manuscript), or elaborate on this item by providing additional information not in the ms, or briefly explain why the item is not applicable/relevant for your study

The intervention does not include any additional non-web-based components or co-interventions (such as telephone support, home programs, or supplementary educational materials). It is a stand-alone, in-person equine-assisted physiotherapy program, which is already clearly reflected in the title.

1a-iii) Primary condition or target group in the title

Mention primary condition or target group in the title, if any (e.g., “for children with Type I Diabetes”) Example: A Web-based and Mobile Intervention with Telephone Support for Children with Type I Diabetes: Randomized Controlled Trial

	1	2	3	4	5	
subitem not at all important	○	○	○	○	◉	essential

Zrušit výběr

Does your paper address subitem 1a-iii? *

Copy and paste relevant sections from manuscript title (include quotes in quotation marks "like this" to indicate direct quotes from your manuscript), or elaborate on this item by providing additional information not in the ms, or briefly explain why the item is not applicable/relevant for your study

Yes.
The primary condition and target group are explicitly stated in the manuscript title.

Relevant excerpt from the title:
"… in Children with Spinal Muscular Atrophy"

1b) ABSTRACT: Structured summary of trial design, methods, results, and conclusions

NPT extension: Description of experimental treatment, comparator, care providers, centers, and blinding status.

1b-i) Key features/functionalities/components of the intervention and comparator in the METHODS section of the ABSTRACT

Mention key features/functionalities/components of the intervention and comparator in the abstract. If possible, also mention theories and principles used for designing the site. Keep in mind the needs of systematic reviewers and indexers by including important synonyms. (Note: Only report in the abstract what the main paper is reporting. If this information is missing from the main body of text, consider adding it)

	1	2	3	4	5	
subitem not at all important	○	○	○	◉	○	essential

Zrušit výběr

Does your paper address subitem 1b-i? *

Copy and paste relevant sections from the manuscript abstract (include quotes in quotation marks "like this" to indicate direct quotes from your manuscript), or elaborate on this item by providing additional information not in the ms, or briefly explain why the item is not applicable/relevant for your study

Yes.
The abstract clearly describes the key components of both the intervention (NEUROEQUIP-SMA) and the comparator (SMA-SOC-N).

Relevant excerpts from the manuscript abstract:

Description of the intervention and comparator:
“The aim of the study is to assess the efficacy of NEUROEQUIP-SMA (A) compared with standard physiotherapy of SMA-SOC-N (B)....”
Key components of intervention and comparator:
“Therapy A consists of a ne... inhibition’ principles (30 min once daily). Both programs include therapeutic horse grooming (20 min a day).”
Primary methodological details:
“In this randomized crossover-controlled t..... sequence.”

These passages identify the main therapeutic principles, the core components of each approach, and the comparative structure of the trial, fully addressing subitem 1b-i.

1b-ii) Level of human involvement in the METHODS section of the ABSTRACT

Clarify the level of human involvement in the abstract, e.g., use phrases like “fully automated” vs. “therapist/nurse/care provider/physician-assisted” (mention number and expertise of providers involved, if any). (Note: Only report in the abstract what the main paper is reporting. If this information is missing from the main body of text, consider adding it)

	1	2	3	4	5	
subitem not at all important	○	○	○	◉	○	essential

Zrušit výběr

Does your paper address subitem 1b-ii?

Copy and paste relevant sections from the manuscript abstract (include quotes in quotation marks "like this" to indicate direct quotes from your manuscript), or elaborate on this item by providing additional information not in the ms, or briefly explain why the item is not applicable/relevant for your study

Partly.
The abstract implies human involvement through the description of physiotherapy sessions but does not explicitly state that the intervention is therapist-delivered or identify the providers involved.
Relevant excerpt:
“Therapy A consists of a newly developed equine-assisted physiotherapy based on neuroproprioceptive ‘facilitation and inhibition’ principles… whereas therapy B involves standard individual outpatient physiotherapy…”
These descriptions indicate that the intervention and comparator require active participation of trained physiotherapists, but the abstract does not explicitly specify the level of human involvement (e.g., “therapist-delivered”), nor does it mention the number or expertise of providers.
Therefore, this item is only indirectly addressed.

1b-iii) Open vs. closed, web-based (self-assessment) vs. face-to-face assessments in the METHODS section of the ABSTRACT

Mention how participants were recruited (online vs. offline), e.g., from an open access website or from a clinic or a closed online user group (closed usergroup trial), and clarify if this was a purely web-based trial, or there were face-to-face components (as part of the intervention or for assessment). Clearly say if outcomes were self-assessed through questionnaires (as common in web-based trials). Note: In traditional offline trials, an open trial (open-label trial) is a type of clinical trial in which both the researchers and participants know which treatment is being administered. To avoid confusion, use “blinded” or “unblinded” to indicated the level of blinding instead of “open”, as “open” in web-based trials usually refers to “open access” (i.e. participants can self-enrol). (Note: Only report in the abstract what the main paper is reporting. If this information is missing from the main body of text, consider adding it)

	1	2	3	4	5	
subitem not at all important	○	○	◉	○	○	essential

Zrušit výběr

Does your paper address subitem 1b-iii?

Copy and paste relevant sections from the manuscript abstract (include quotes in quotation marks "like this" to indicate direct quotes from your manuscript), or elaborate on this item by providing additional information not in the ms, or briefly explain why the item is not applicable/relevant for your study

Partly.
The abstract describes a randomized crossover-controlled clinical trial conducted in person, but it does not explicitly state that the trial is fully face-to-face, nor does it clarify recruitment mode or specify that no web-based self-assessments were used.
Relevant excerpts:
“…20 children with spinal muscular atrophy… will participate in two six-day therapy programs (A and B)…”
This indicates in-person participation and clinic-based procedures, but the abstract does not explicitly state that recruitment was offline (clinic-based) or that all assessments were performed face-to-face by trained physiotherapists, nor does it clarify that no web-based components or self-assessment tools were used.

Therefore, the item is only indirectly addressed.

1b-iv) RESULTS section in abstract must contain use data

Report number of participants enrolled/assessed in each group, the use/uptake of the intervention (e.g., attrition/adherence metrics, use over time, number of logins etc.), in addition to primary/secondary outcomes. (Note: Only report in the abstract what the main paper is reporting. If this information is missing from the main body of text, consider adding it)

	1	2	3	4	5	
subitem not at all important	◉	○	○	○	○	essential

Zrušit výběr

Does your paper address subitem 1b-iv?

Copy and paste relevant sections from the manuscript abstract (include quotes in quotation marks "like this" to indicate direct quotes from your manuscript), or elaborate on this item by providing additional information not in the ms, or briefly explain why the item is not applicable/relevant for your study

Not applicable.
This protocol does not report results yet, as no participants have been enrolled and the study has not started data collection. Therefore, the abstract does not contain use data, adherence information, or attrition metrics.

Relevant excerpt from the abstract:
“The study is designed for 20 participants. Data collection will begin in February 2026 and will be completed in May 2026. Data analyses are planned for autumn 2026, and study results are expected to be available in 2027.”

1b-v) CONCLUSIONS/DISCUSSION in abstract for negative trials

Conclusions/Discussions in abstract for negative trials: Discuss the primary outcome - if the trial is negative (primary outcome not changed), and the intervention was not used, discuss whether negative results are attributable to lack of uptake and discuss reasons. (Note: Only report in the abstract what the main paper is reporting. If this information is missing from the main body of text, consider adding it)

	1	2	3	4	5	
subitem not at all important	(x)	( )	( )	( )	( )	essential

Zrušit výběr

Does your paper address subitem 1b-v?

Copy and paste relevant sections from the manuscript abstract (include quotes in quotation marks "like this" to indicate direct quotes from your manuscript), or elaborate on this item by providing additional information not in the ms, or briefly explain why the item is not applicable/relevant for your study

Not applicable.
This manuscript is a study protocol, and therefore no trial results—positive or negative—are available yet.

INTRODUCTION

2a) In INTRODUCTION: Scientific background and explanation of rationale

2a-i) Problem and the type of system/solution

Describe the problem and the type of system/solution that is object of the study: intended as stand-alone intervention vs. incorporated in broader health care program? Intended for a particular patient population? Goals of the intervention, e.g., being more cost-effective to other interventions, replace or complement other solutions? (Note: Details about the intervention are provided in “Methods” under 5)

	1	2	3	4	5	
subitem not at all important	○	○	○	○	◉	essential

Zrušit výběr

Does your paper address subitem 2a-i? *

Copy and paste relevant sections from the manuscript (include quotes in quotation marks "like this" to indicate direct quotes from your manuscript), or elaborate on this item by providing additional information not in the ms, or briefly explain why the item is not applicable/relevant for your study

Yes.
The manuscript clearly describes the clinical problem, the target population, and the type and purpose of the intervention.
The clinical problem and its relevance are outlined in the Introduction, e.g.:
“Spinal muscular atrophy (SMA) is a rare neuromuscular disease and the most common genetic cause of infant death.”
“Although pharmacological treatment improves survival rates and functional capacity, physiotherapy remains a key component of care.”

“Despite advances in gene and molecular therapies, there remains a critical need for complementary rehabilitation approaches that can enhance motor function, postural control, breathing patterns, and overall quality of life.”

2a-ii) Scientific background, rationale: What is known about the (type of) system

Scientific background, rationale: What is known about the (type of) system that is the object of the study (be sure to discuss the use of similar systems for other conditions/diagnoses, if appropiate), motivation for the study, i.e. what are the reasons for and what is the context for this specific study, from which stakeholder viewpoint is the study performed, potential impact of findings [2]. Briefly justify the choice of the comparator.

	1	2	3	4	5	
subitem not at all important	○	○	○	○	◉	essential

Zrušit výběr

Does your paper address subitem 2a-ii? *

Copy and paste relevant sections from the manuscript (include quotes in quotation marks "like this" to indicate direct quotes from your manuscript), or elaborate on this item by providing additional information not in the ms, or briefly explain why the item is not applicable/relevant for your study

Yes. The manuscript provides a comprehensive scientific background and clear rationale for the study, including what is known about SMA, physiotherapy approaches, equine-assisted physiotherapy, and neuroproprioceptive “facilitation and inhibition.” It also explains the motivation for developing the new intervention and justifies the choice of comparator.

Relevant sections from the manuscript include:

Scientific background on SMA and physiotherapy:
“Spinal muscular atrophy (SMA) is an autosomal recessive neuromuscular disorder… multidisciplinary supportive care remains essential to achieve optimal outcomes, with physiotherapy (PT) being an integral part of it.”
“The perspective on PT has been rapidly evolving… Nowadays, the focus has shifted more towards enhancing functional mobility.”

Rationale for developing the new system/intervention:
“This new insight into the motor units’ adaptations significantly influenced and motivated us to develop a new active physiotherapy approach for children with SMA.”

Description of what is known about similar systems:
“Standard Equine-Assisted Physiotherapy has the potential to influence multiple domains, including posture, balance, and coordination… In children with SMA, it leads to physical improvements… and psychological improvements…”

Clear definition of the system under study:
“We have developed a unique method of Equine-Assisted Physiotherapy Based on Neuroproprioceptive ‘Facilitation and Inhibition’ (NEUROEQUIP-SMA).”

Rationale for the comparator:
“Therapy follows the recommendations of the International Coordinating Committee for SMA Clinical Trials – Standard of Care (SMA-SOC).”
“Their inclusion also allows for a better comparison of the effect of the active comparator with the intervention group, as both approaches will work with a similar degree of ‘facilitation and inhibition’ within motor patterns.”

Context, motivation, expected impact:
“This study may be the first step towards understanding the involvement of lncRNAs in the pathophysiology of this disease and their use as prognostic markers.”

2b) In INTRODUCTION: Specific objectives or hypotheses

Does your paper address CONSORT subitem 2b? *

Copy and paste relevant sections from the manuscript (include quotes in quotation marks "like this" to indicate direct quotes from your manuscript), or elaborate on this item by providing additional information not in the ms, or briefly explain why the item is not applicable/relevant for your study

Yes. The manuscript clearly states the primary and secondary objectives as well as the corresponding hypotheses.

Relevant sections from the manuscript:

Objectives:

“The primary objective is to evaluate the immediate effect of a six-day intervention on muscle fatigue, coordination and postural control, quality and quantity of psychomotor development, respiratory function, and development/progression of scoliotic posture.”

“The secondary goal is to monitor the long-term effect of the intervention so that it is clinically relevant for the client and at the same time as persistent as possible.”

Primary hypotheses:

“Immediately after the six-day intensive program, we expect improvements in both groups; however, we anticipate more pronounced effects in NEUROEQUIP-SMA…” followed by specific domains such as: “No undesirable muscle fatigue (assessed using surface electromyography, sEMG). Improved coordination of the torso and cervical spine… Same or improved scores on the Hammersmith Functional Motor Scale… Improved spirometry and scoliometric measurements. Improved performance in the Segmental Assessment of Trunk Control, Trunk Control Measurement Scale, Modified Functional Reach Test and Selective Control Assessment of the Lower Extremity.”

Secondary hypothesis:

“We anticipate improvements in both groups; however, NEUROEQUIP-SMA is expected to demonstrate a greater enhancement in quality of life… as well as a more substantial increase in both the quality and scope of psychomotor development… Moreover, molecular biological markers are expected to change immediately [after] the completion of the therapeutic program.”

METHODS

3a) Description of trial design (such as parallel, factorial) including allocation ratio

Does your paper address CONSORT subitem 3a? *

Copy and paste relevant sections from the manuscript (include quotes in quotation marks "like this" to indicate direct quotes from your manuscript), or elaborate on this item by providing additional information not in the ms, or briefly explain why the item is not applicable/relevant for your study

Yes.
Our manuscript clearly describes the trial design, including the type of trial, allocation ratio, and key structural elements of the design.

Relevant sections from the manuscript include:

Description of trial design:
“This study is designed as a single-blind randomized crossover-controlled trial.”

Allocation ratio and structure:
“Participants will be randomized in a 1:1 ratio to receive both therapies in either the AB or BA sequence, over two periods separated by a 10 weeks washout period.”

Duration and rationale for period length:
“Every child will undergo two six-day therapeutic programs, administered in two separate periods.”

“The six-day duration of each period was chosen to capture the immediate (short-term) response.”

Additional rationale for design choice:
“The short, intensive window allows for standardized application, limits the influence of concurrent rehabilitation, and allows each participant to act as their own control.”

3b) Important changes to methods after trial commencement (such as eligibility criteria), with reasons

Does your paper address CONSORT subitem 3b? *

Copy and paste relevant sections from the manuscript (include quotes in quotation marks "like this" to indicate direct quotes from your manuscript), or elaborate on this item by providing additional information not in the ms, or briefly explain why the item is not applicable/relevant for your study

Not applicable.
This manuscript is a study protocol, and therefore no changes to methods after trial commencement have occurred.

3b-i) Bug fixes, Downtimes, Content Changes

Bug fixes, Downtimes, Content Changes: ehealth systems are often dynamic systems. A description of changes to methods therefore also includes important changes made on the intervention or comparator during the trial (e.g., major bug fixes or changes in the functionality or content) (5-iii) and other “unexpected events” that may have influenced study design such as staff changes, system failures/downtimes, etc. [2].

	1	2	3	4	5	
subitem not at all important	(●)	( )	( )	( )	( )	essential

Zrušit výběr

Does your paper address subitem 3b-i?

Copy and paste relevant sections from the manuscript (include quotes in quotation marks "like this" to indicate direct quotes from your manuscript), or elaborate on this item by providing additional information not in the ms, or briefly explain why the item is not applicable/relevant for your study

Not applicable.
This study does not use any web-based, mobile, or electronic system. The intervention is an in-person physiotherapy program (equine-assisted, neuroproprioceptive), and therefore no software updates, bug fixes, content modifications, or system downtimes could occur.

Additionally, as this is a study protocol and data collection has not yet begun, no unexpected events (e.g., staff changes, system failures) have occurred that could influence the study design.

4a) Eligibility criteria for participants

Does your paper address CONSORT subitem 4a? *

Copy and paste relevant sections from the manuscript (include quotes in quotation marks "like this" to indicate direct quotes from your manuscript), or elaborate on this item by providing additional information not in the ms, or briefly explain why the item is not applicable/relevant for your study

Yes.
The manuscript clearly describes the eligibility criteria for participants, including diagnosis, age range, treatment status, and clinical requirements.

Relevant sections from the manuscript:

Inclusion criteria:
“Participants will be children with SMA types I, II, and III, aged two to nine years.”
“Participants must be receiving gene/substitution therapy (nusinersen, onasemnogene, or risdiplam), be clinically stable, and able to safely participate in equine-assisted physiotherapy.”

Exclusion criteria:
„Exclusion criteria will include hip subluxation, allergies to horses or the stable environment, and an overwhelming fear of horses and no intensive therapy 10 weeks before study intervention.“

4a-i) Computer / Internet literacy

Computer / Internet literacy is often an implicit “de facto” eligibility criterion - this should be explicitly clarified.

	1	2	3	4	5	
subitem not at all important	(●)	○	○	○	○	essential

Zrušit výběr

Does your paper address subitem 4a-i?

Copy and paste relevant sections from the manuscript (include quotes in quotation marks "like this" to indicate direct quotes from your manuscript), or elaborate on this item by providing additional information not in the ms, or briefly explain why the item is not applicable/relevant for your study

Not applicable.
This study does not involve any web-based, mobile, or computer-based intervention and does not require participants to use digital technologies.

4a-ii) Open vs. closed, web-based vs. face-to-face assessments:

Open vs. closed, web-based vs. face-to-face assessments: Mention how participants were recruited (online vs. offline), e.g., from an open access website or from a clinic, and clarify if this was a purely web-based trial, or there were face-to-face components (as part of the intervention or for assessment), i.e., to what degree got the study team to know the participant. In online-only trials, clarify if participants were quasi-anonymous and whether having multiple identities was possible or whether technical or logistical measures (e.g., cookies, email confirmation, phone calls) were used to detect/prevent these.

	1	2	3	4	5	
subitem not at all important	○	◉	○	○	○	essential

Zrušit výběr

Does your paper address subitem 4a-ii? *

Copy and paste relevant sections from the manuscript (include quotes in quotation marks "like this" to indicate direct quotes from your manuscript), or elaborate on this item by providing additional information not in the ms, or briefly explain why the item is not applicable/relevant for your study

Yes. The manuscript clearly describes that this is an offline, clinic-based, face-to-face clinical trial, not a web-based or online study. Participants were not recruited online, and all assessments as well as interventions were conducted in person.

Recruitment is described as follows:
“Participants will be simultaneously recruited from the database of the patient organization SMÁci, z. s., which registers 90% of children with SMA in the Czech Republic, while the remaining participants will be sourced from the database of the Mirákl Hippotherapy Center, o.p.s. All parents of children who meet the entry criteria will be contacted.”

4a-iii) Information giving during recruitment

Information given during recruitment. Specify how participants were briefed for recruitment and in the informed consent procedures (e.g., publish the informed consent documentation as appendix, see also item X26), as this information may have an effect on user self-selection, user expectation and may also bias results.

	1	2	3	4	5	
subitem not at all important	○	○	◉	○	○	essential

Zrušit výběr

Does your paper address subitem 4a-iii?

Copy and paste relevant sections from the manuscript (include quotes in quotation marks "like this" to indicate direct quotes from your manuscript), or elaborate on this item by providing additional information not in the ms, or briefly explain why the item is not applicable/relevant for your study

Yes.
The manuscript clearly describes how participants and their parents/legal guardians were informed during recruitment and how the informed consent process was conducted.

Relevant sections from the manuscript:

Description of the informed consent process:
"Parents or legal representatives of all children will receive detailed information about the study, including its purpose, procedures, potential benefits, and risks. Written informed consent will be obtained from all parents/legal guardians prior to participation."

Information provided during recruitment:
"All parents of children who meet the entry criteria will be contacted. They will be fully informed about the study procedures, timing, and the nature of both therapeutic programs before agreeing to participate."

Ethics approval and transparency:
"The study was approved by the Ethics Committee of the Third Faculty of Medicine, Charles University, and the Thomayer University Hospital (Ref: 115/2024)."

This demonstrates that standard ethical procedures for participant information and consent were followed.

4b) Settings and locations where the data were collected

Does your paper address CONSORT subitem 4b? *

Copy and paste relevant sections from the manuscript (include quotes in quotation marks "like this" to indicate direct quotes from your manuscript), or elaborate on this item by providing additional information not in the ms, or briefly explain why the item is not applicable/relevant for your study

Yes, our manuscript addresses this item.
The study setting is explicitly described in the METHODS section. Relevant text from the manuscript includes:

“Clinical examinations will be conducted at the College of Polytechnics Jihlava, which is equipped with advanced technology, including a 16-channel surface EMG system (DELSYS), spirometry, and Qualisys Motion Capture System with 16 cameras.”

“The Mirákl Hippotherapy Center, a healthcare facility offering year-round Equine-Assisted Physiotherapy programs for children with special needs, has extensive experience and resources to support the study.”

“LncRNA analysis will be performed at the Department of Medical Genetics, Third Faculty of Medicine, Charles University, which houses a state-of-the-art molecular biology laboratory specializing in genomic and biomarker research.”

4b-i) Report if outcomes were (self-)assessed through online questionnaires

Clearly report if outcomes were (self-)assessed through online questionnaires (as common in web-based trials) or otherwise.

	1	2	3	4	5	
subitem not at all important	○	○	◉	○	○	essential

Zrušit výběr

Does your paper address subitem 4b-i? *

Copy and paste relevant sections from the manuscript (include quotes in quotation marks "like this" to indicate direct quotes from your manuscript), or elaborate on this item by providing additional information not in the ms, or briefly explain why the item is not applicable/relevant for your study

Yes, the paper addresses this subitem.
„Parents of children with SMA will complete the PedsQL™ Generic Core Scales – Parent Proxy Report.“

„Parents of children with SMA will also complete the Strengths and Difficulties Questionnaire (SDQ).“

„Parents… will complete a special questionnaire at the beginning and 28 days after the end of the rehabilitation.“ (ICF)

These outcomes are therefore self-reported by parents, but they are not collected through online questionnaires. Instead, they are administered and collected as part of the scheduled assessment procedures described in the study protocol.

4b-ii) Report how institutional affiliations are displayed

Report how institutional affiliations are displayed to potential participants [on ehealth media], as affiliations with prestigious hospitals or universities may affect volunteer rates, use, and reactions with regards to an intervention.(Not a required item – describe only if this may bias results)

	1	2	3	4	5	
subitem not at all important	(●)	( )	( )	( )	( )	essential

Zrušit výběr

Does your paper address subitem 4b-ii?

Copy and paste relevant sections from the manuscript (include quotes in quotation marks "like this" to indicate direct quotes from your manuscript), or elaborate on this item by providing additional information not in the ms, or briefly explain why the item is not applicable/relevant for your study

Not applicable.
This study does not use any web-based platform, online recruitment, or electronic media where institutional affiliations might be displayed to potential participants. Recruitment occurs through direct contact with families via the patient organization SMÁci and the Mirákl Hippotherapy Center, and participation is based on standard clinical communication rather than online presentation.

5) The interventions for each group with sufficient details to allow replication, including how and when they were actually administered

5-i) Mention names, credential, affiliations of the developers, sponsors, and owners

Mention names, credential, affiliations of the developers, sponsors, and owners [6] (if authors/evaluators are owners or developer of the software, this needs to be declared in a “Conflict of interest” section or mentioned elsewhere in the manuscript).

	1	2	3	4	5	
subitem not at all important	(●)	( )	( )	( )	( )	essential

Zrušit výběr

Does your paper address subitem 5-i?

Copy and paste relevant sections from the manuscript (include quotes in quotation marks "like this" to indicate direct quotes from your manuscript), or elaborate on this item by providing additional information not in the ms, or briefly explain why the item is not applicable/relevant for your study

Not applicable.
This study does not involve any software, web-based platform, mobile application, or digital system. The intervention evaluated in this trial is a physiotherapeutic method (NEUROEQUIP-SMA), not an eHealth product. Therefore, concepts such as software developers, system owners, or digital sponsors do not apply.

5-ii) Describe the history/development process

Describe the history/development process of the application and previous formative evaluations (e.g., focus groups, usability testing), as these will have an impact on adoption/use rates and help with interpreting results.

	1	2	3	4	5	
subitem not at all important	◉	○	○	○	○	essential

Zrušit výběr

Does your paper address subitem 5-ii?

Copy and paste relevant sections from the manuscript (include quotes in quotation marks "like this" to indicate direct quotes from your manuscript), or elaborate on this item by providing additional information not in the ms, or briefly explain why the item is not applicable/relevant for your study

Not applicable.
This study does not evaluate a digital system or application. The intervention is a physiotherapeutic method (NEUROEQUIP-SMA), not an eHealth product. Therefore, this item—focused on the development history, iterative testing, or usability evaluation of software—does not apply.

5-iii) Revisions and updating

Revisions and updating. Clearly mention the date and/or version number of the application/intervention (and comparator, if applicable) evaluated, or describe whether the intervention underwent major changes during the evaluation process, or whether the development and/or content was “frozen” during the trial. Describe dynamic components such as news feeds or changing content which may have an impact on the replicability of the intervention (for unexpected events see item 3b).

	1	2	3	4	5	
subitem not at all important	◉	○	○	○	○	essential

Zrušit výběr

Does your paper address subitem 5-iii?

Copy and paste relevant sections from the manuscript (include quotes in quotation marks "like this" to indicate direct quotes from your manuscript), or elaborate on this item by providing additional information not in the ms, or briefly explain why the item is not applicable/relevant for your study

Not applicable.
The intervention evaluated in this study is a physiotherapeutic method (NEUROEQUIP-SMA), not a software application or digital platform. Therefore, concepts such as version numbers, application updates, dynamic content, or ongoing revisions do not apply. The therapeutic approach was defined in the study protocol and remained stable for the purposes of the trial, but no digital system exists to which this subitem would refer.

5-iv) Quality assurance methods

Provide information on quality assurance methods to ensure accuracy and quality of information provided [1], if applicable.

	1	2	3	4	5	
subitem not at all important	◉	○	○	○	○	essential

Zrušit výběr

Does your paper address subitem 5-iv?

Copy and paste relevant sections from the manuscript (include quotes in quotation marks "like this" to indicate direct quotes from your manuscript), or elaborate on this item by providing additional information not in the ms, or briefly explain why the item is not applicable/relevant for your study

Not applicable.
This study does not involve a web-based system, digital platform, or electronic content that would require information quality assurance procedures.

5-v) Ensure replicability by publishing the source code, and/or providing screenshots/screen-capture video, and/or providing flowcharts of the algorithms used

Ensure replicability by publishing the source code, and/or providing screenshots/screen-capture video, and/or providing flowcharts of the algorithms used. Replicability (i.e., other researchers should in principle be able to replicate the study) is a hallmark of scientific reporting.

	1	2	3	4	5	
subitem not at all important	◉	○	○	○	○	essential

Zrušit výběr

Does your paper address subitem 5-v?

Copy and paste relevant sections from the manuscript (include quotes in quotation marks "like this" to indicate direct quotes from your manuscript), or elaborate on this item by providing additional information not in the ms, or briefly explain why the item is not applicable/relevant for your study

Not applicable.
This study does not involve any software, web-based system, mobile application, or digital platform.

5-vi) Digital preservation

Digital preservation: Provide the URL of the application, but as the intervention is likely to change or disappear over the course of the years; also make sure the intervention is archived (Internet Archive, webcitation.org, and/or publishing the source code or screenshots/videos alongside the article). As pages behind login screens cannot be archived, consider creating demo pages which are accessible without login.

	1	2	3	4	5	
subitem not at all important	◉	○	○	○	○	essential

Zrušit výběr

Does your paper address subitem 5-vi?

Copy and paste relevant sections from the manuscript (include quotes in quotation marks "like this" to indicate direct quotes from your manuscript), or elaborate on this item by providing additional information not in the ms, or briefly explain why the item is not applicable/relevant for your study

Not applicable.
This study does not use any digital application, web-based system, or mobile platform.

5-vii) Access

Access: Describe how participants accessed the application, in what setting/context, if they had to pay (or were paid) or not, whether they had to be a member of specific group. If known, describe how participants obtained “access to the platform and Internet” [1]. To ensure access for editors/reviewers/readers, consider to provide a “backdoor” login account or demo mode for reviewers/readers to explore the application (also important for archiving purposes, see vi).

	1	2	3	4	5	
subitem not at all important	◉	○	○	○	○	essential

Zrušit výběr

Does your paper address subitem 5-vii? *

Copy and paste relevant sections from the manuscript (include quotes in quotation marks "like this" to indicate direct quotes from your manuscript), or elaborate on this item by providing additional information not in the ms, or briefly explain why the item is not applicable/relevant for your study

Not applicable.
This study does not use any digital application, web-based system, or mobile platform.

5-viii) Mode of delivery, features/functionalities/components of the intervention and comparator, and the theoretical framework

Describe mode of delivery, features/functionalities/components of the intervention and comparator, and the theoretical framework [6] used to design them (instructional strategy [1], behaviour change techniques, persuasive features, etc., see e.g., [7, 8] for terminology). This includes an in-depth description of the content (including where it is coming from and who developed it) [1],” whether [and how] it is tailored to individual circumstances and allows users to track their progress and receive feedback” [6]. This also includes a description of communication delivery channels and – if computer-mediated communication is a component – whether communication was synchronous or asynchronous [6]. It also includes information on presentation strategies [1], including page design principles, average amount of text on pages, presence of hyperlinks to other resources, etc. [1].

	1	2	3	4	5	
subitem not at all important	(•)	( )	( )	( )	( )	essential

Zrušit výběr

Does your paper address subitem 5-viii? *

Copy and paste relevant sections from the manuscript (include quotes in quotation marks "like this" to indicate direct quotes from your manuscript), or elaborate on this item by providing additional information not in the ms, or briefly explain why the item is not applicable/relevant for your study

Not applicable.
This study does not involve any web-based, mobile, or digital intervention. The evaluated intervention (NEUROEQUIP-SMA) is an in-person physiotherapeutic method delivered by trained physiotherapists.

5-ix) Describe use parameters

Describe use parameters (e.g., intended “doses” and optimal timing for use). Clarify what instructions or recommendations were given to the user, e.g., regarding timing, frequency, heaviness of use, if any, or was the intervention used ad libitum.

	1	2	3	4	5	
subitem not at all important	(●)	( )	( )	( )	( )	essential

Zrušit výběr

Does your paper address subitem 5-ix?

Copy and paste relevant sections from the manuscript (include quotes in quotation marks "like this" to indicate direct quotes from your manuscript), or elaborate on this item by providing additional information not in the ms, or briefly explain why the item is not applicable/relevant for your study

Not applicable.
This study does not involve any web-based, mobile, or digital intervention. The evaluated intervention (NEUROEQUIP-SMA) is an in-person physiotherapeutic method delivered by trained physiotherapists.

5-x) Clarify the level of human involvement

Clarify the level of human involvement (care providers or health professionals, also technical assistance) in the e-intervention or as co-intervention (detail number and expertise of professionals involved, if any, as well as “type of assistance offered, the timing and frequency of the support, how it is initiated, and the medium by which the assistance is delivered”. It may be necessary to distinguish between the level of human involvement required for the trial, and the level of human involvement required for a routine application outside of a RCT setting (discuss under item 21 – generalizability).

	1	2	3	4	5	
subitem not at all important	( )	( )	( )	( )	(●)	essential

Zrušit výběr

Does your paper address subitem 5-x?

Copy and paste relevant sections from the manuscript (include quotes in quotation marks "like this" to indicate direct quotes from your manuscript), or elaborate on this item by providing additional information not in the ms, or briefly explain why the item is not applicable/relevant for your study

NEUROEQUIP-SMA is delivered exclusively in person by highly trained physiotherapists, and the protocol specifies their qualifications, training, supervision, and role in the intervention.

Relevant manuscript sections include:

“The intervention is performed by a physiotherapist who is specially trained and certified in equine-assisted physiotherapy…”
“…working under professional supervision.”
“All horses and physiotherapists belong to the Hippotherapy Center Mirákl, which provides a standardized therapeutic environment.”

5-xi) Report any prompts/reminders used

Report any prompts/reminders used: Clarify if there were prompts (letters, emails, phone calls, SMS) to use the application, what triggered them, frequency etc. It may be necessary to distinguish between the level of prompts/reminders required for the trial, and the level of prompts/reminders for a routine application outside of a RCT setting (discuss under item 21 – generalizability).

	1	2	3	4	5	
subitem not at all important	◉	○	○	○	○	essential

Zrušit výběr

Does your paper address subitem 5-xi? *

Copy and paste relevant sections from the manuscript (include quotes in quotation marks "like this" to indicate direct quotes from your manuscript), or elaborate on this item by providing additional information not in the ms, or briefly explain why the item is not applicable/relevant for your study

Not applicable.
This study does not involve any web-based or mobile application, and no digital prompts or reminders (e.g., emails, SMS, app notifications) were used.

5-xii) Describe any co-interventions (incl. training/support)

Describe any co-interventions (incl. training/support): Clearly state any interventions that are provided in addition to the targeted eHealth intervention, as ehealth intervention may not be designed as stand-alone intervention. This includes training sessions and support [1]. It may be necessary to distinguish between the level of training required for the trial, and the level of training for a routine application outside of a RCT setting (discuss under item 21 – generalizability.

	1	2	3	4	5	
subitem not at all important	○	○	○	○	◉	essential

Zrušit výběr

Does your paper address subitem 5-xii? *

Copy and paste relevant sections from the manuscript (include quotes in quotation marks "like this" to indicate direct quotes from your manuscript), or elaborate on this item by providing additional information not in the ms, or briefly explain why the item is not applicable/relevant for your study

Yes.
The manuscript clearly describes the presence or absence of co-interventions. For the intervention group, no additional therapies are allowed during the 6-day intensive NEUROEQUIP-SMA program, and no intensive rehabilitation is permitted for 4–6 weeks afterwards:

“During the intensive 6-day program, no other physiotherapy or rehabilitation interventions are permitted.”

“For 4–6 weeks after the intervention, participants do not undergo any intensive rehabilitation.”

For the control group, the protocol states that they will receive standard physiotherapy:

“The control group will receive conventional physiotherapy as indicated by their standard care plan.”

6a) Completely defined pre-specified primary and secondary outcome measures, including how and when they were assessed

Does your paper address CONSORT subitem 6a? *

Copy and paste relevant sections from the manuscript (include quotes in quotation marks "like this" to indicate direct quotes from your manuscript), or elaborate on this item by providing additional information not in the ms, or briefly explain why the item is not applicable/relevant for your study

Yes.
The manuscript clearly specifies all primary and secondary outcome measures, including their definitions, timing of assessment, procedures, and interpretation. The primary outcomes are motor coordination (3D motion analysis), muscle fatigue (sEMG), respiratory function (spirometry), standardized motor scales, clinical tests of trunk and cervical endurance, and scoliometer measurements. Secondary outcomes include quality-of-life assessments, home-video analysis of psychomotor development, and molecular biomarkers (lncRNA expression from blood samples). All outcomes are described in sufficient detail to allow replication, including measurement protocols, equipment used, marker placement, scoring procedures, and timing of assessments (baseline, post-intervention, and day-28 follow-up).

6a-i) Online questionnaires: describe if they were validated for online use and apply CHERRIES items to describe how the questionnaires were designed/deployed

If outcomes were obtained through online questionnaires, describe if they were validated for online use and apply CHERRIES items to describe how the questionnaires were designed/deployed [9].

	1	2	3	4	5	
subitem not at all important	◉	○	○	○	○	essential

Zrušit výběr

Does your paper address subitem 6a-i?

Copy and paste relevant sections from manuscript text

No outcomes in this study are obtained through online questionnaires.
All questionnaires used in the trial (e.g., PedsQL™, SDQ) are administered in person or via paper-based completion during clinical assessments. As no data are collected through web-based or electronic survey platforms, validation for online use is not applicable, and CHERRIES reporting items do not apply to this study.

6a-ii) Describe whether and how “use” (including intensity of use/dosage) was defined/measured/monitored

Describe whether and how “use” (including intensity of use/dosage) was defined/measured/monitored (logins, logfile analysis, etc.). Use/adoption metrics are important process outcomes that should be reported in any ehealth trial.

	1	2	3	4	5	
subitem not at all important	○	○	○	○	◉	essential

Zrušit výběr

Does your paper address subitem 6a-ii?

Copy and paste relevant sections from manuscript text

Yes. Relevant sections from the manuscript text:
"Primary outcome measures

3D Motion-Based Evaluation of Postural Control, Breathing, and Coordination

All three tests share a common setup, in which the child sits straddling a cylinder with sensors attached. The cylinder is positioned within a calibrated 3D space and firmly secured to prevent movement" etc.

6a-iii) Describe whether, how, and when qualitative feedback from participants was obtained

Describe whether, how, and when qualitative feedback from participants was obtained (e.g., through emails, feedback forms, interviews, focus groups).

	1	2	3	4	5	
subitem not at all important	◉	○	○	○	○	essential

Zrušit výběr

Does your paper address subitem 6a-iii?

Copy and paste relevant sections from manuscript text

Not applicable.
The study does not collect qualitative feedback from participants or parents, such as interviews, focus groups, open-ended questions, or narrative comments.

6b) Any changes to trial outcomes after the trial commenced, with reasons

Does your paper address CONSORT subitem 6b? *

Copy and paste relevant sections from the manuscript (include quotes in quotation marks "like this" to indicate direct quotes from your manuscript), or elaborate on this item by providing additional information not in the ms, or briefly explain why the item is not applicable/relevant for your study

Not applicable.
This manuscript reports a study protocol. The trial has not yet commenced, and therefore no changes to outcome measures after trial initiation have occurred.

7a) How sample size was determined

NPT: When applicable, details of whether and how the clustering by care provides or centers was addressed

7a-i) Describe whether and how expected attrition was taken into account when calculating the sample size

Describe whether and how expected attrition was taken into account when calculating the sample size.

	1	2	3	4	5	
subitem not at all important	○	○	◉	○	○	essential

Zrušit výběr

Does your paper address subitem 7a-i?

Copy and paste relevant sections from manuscript title (include quotes in quotation marks "like this" to indicate direct quotes from your manuscript), or elaborate on this item by providing additional information not in the ms, or briefly explain why the item is not applicable/relevant for your study

Partly.

Explanation:
The sample size was calculated based on the expected detectable effect size and statistical power; however, anticipated attrition was not explicitly incorporated into the sample size calculation. Recruitment aims to include all eligible children in this rare-disease population (SMA), which limits the possibility of inflating the sample size to compensate for dropout.

7b) When applicable, explanation of any interim analyses and stopping guidelines

Does your paper address CONSORT subitem 7b? *

Copy and paste relevant sections from the manuscript (include quotes in quotation marks "like this" to indicate direct quotes from your manuscript), or elaborate on this item by providing additional information not in the ms, or briefly explain why the item is not applicable/relevant for your study

Not applicable.
The study does not include any interim analyses or stopping guidelines. As described in the protocol, this is a small pilot trial with a fixed sample size and no planned interim data reviews. The trial will run until all participants complete the protocol unless safety concerns arise, in which case standard clinical procedures would apply. No formal early stopping rules are defined.

8a) Method used to generate the random allocation sequence

NPT: When applicable, how care providers were allocated to each trial group

Does your paper address CONSORT subitem 8a? *

Copy and paste relevant sections from the manuscript (include quotes in quotation marks "like this" to indicate direct quotes from your manuscript), or elaborate on this item by providing additional information not in the ms, or briefly explain why the item is not applicable/relevant for your study

Yes.
The manuscript clearly describes how the random allocation sequence is generated. The protocol states that participants will be randomly assigned to the intervention group or the control group using computer-generated randomization. Specifically, the manuscript states:

"Randomization will be performed using computer-generated random numbers."

8b) Type of randomisation; details of any restriction (such as blocking and block size)

Does your paper address CONSORT subitem 8b? *

Copy and paste relevant sections from the manuscript (include quotes in quotation marks "like this" to indicate direct quotes from your manuscript), or elaborate on this item by providing additional information not in the ms, or briefly explain why the item is not applicable/relevant for your study

Yes.
The manuscript describes the type of randomisation and the use of restricted block randomisation with variable block sizes:

"Block randomization with variable block sizes of 2 and 4 will be used to achieve balanced sequence sizes (AB and BA) within each therapeutic cycle of 20 children."

9) Mechanism used to implement the random allocation sequence (such as sequentially numbered containers), describing any steps taken to conceal the sequence until interventions were assigned

Does your paper address CONSORT subitem 9? *

Copy and paste relevant sections from the manuscript (include quotes in quotation marks "like this" to indicate direct quotes from your manuscript), or elaborate on this item by providing additional information not in the ms, or briefly explain why the item is not applicable/relevant for your study

"Random allocation was implemented using opaque, sealed envelopes to ensure concealment of the assignment. The randomization sequence was generated externally and was not accessible to therapists, investigators, or outcome assessors. Envelopes were opened sequentially only after written informed consent was obtained. This procedure ensured that the allocation could not be anticipated before assignment."

"Outcome assessors remained blinded throughout the study. All clinical examinations and instrumental assessments were performed in the same controlled environment regardless of treatment sequence. Due to the nature of the interventions, blinding of therapists, children, and parents was not feasible, but families were instructed not to disclose which therapy the child received."

10) Who generated the random allocation sequence, who enrolled participants, and who assigned participants to interventions

Does your paper address CONSORT subitem 10? *

Copy and paste relevant sections from the manuscript (include quotes in quotation marks "like this" to indicate direct quotes from your manuscript), or elaborate on this item by providing additional information not in the ms, or briefly explain why the item is not applicable/relevant for your study

Partly.
The manuscript describes blinding procedures, but does not fully describe the mechanism used to conceal the random allocation sequence until assignment. Outcome assessment is conducted by a blinded assessor, as the protocol states that a blinded assessor will collect outcome data and will not be informed about the intervention sequence received by each child.

However, the protocol does not specify in detail how the randomisation list itself is concealed from investigators (e.g., sealed, opaque envelopes, central allocation, or an automated system). Therefore, this item is only partly addressed.

11a) If done, who was blinded after assignment to interventions (for example, participants, care providers, those assessing outcomes) and how
NPT: Whether or not administering co-interventions were blinded to group assignment

11a-i) Specify who was blinded, and who wasn't

Specify who was blinded, and who wasn't. Usually, in web-based trials it is not possible to blind the participants [1, 3] (this should be clearly acknowledged), but it may be possible to blind outcome assessors, those doing data analysis or those administering co-interventions (if any).

	1	2	3	4	5	
subitem not at all important	○	○	○	○	◉	essential

Zrušit výběr

Does your paper address subitem 11a-i? *

Copy and paste relevant sections from the manuscript (include quotes in quotation marks "like this" to indicate direct quotes from your manuscript), or elaborate on this item by providing additional information not in the ms, or briefly explain why the item is not applicable/relevant for your study

Outcome assessors and investigators were blinded to group assignment.
The protocol states that:
“Outcome assessors and investigators will remain blinded to minimize potential bias in outcome assessments. All examinations will take place in the same examination room under identical conditions, regardless of the assigned intervention.”
Participants, parents, and therapists could not be blinded due to the nature of the intervention:
“Blinding will not be feasible for therapists, children with SMA, or their parents due to the nature of the interventions.”

To reduce bias, families were instructed not to reveal the assigned therapy:
“Children and parents will be strongly advised not to disclose the allocated therapy during both the baseline and final assessments.”

Co-interventions:
No additional co-interventions are administered by blinded personnel; permitted and prohibited concomitant care is standardized outside the blinded assessment process.

11a-ii) Discuss e.g., whether participants knew which intervention was the "intervention of interest" and which one was the "comparator"

Informed consent procedures (4a-ii) can create biases and certain expectations - discuss e.g., whether participants knew which intervention was the "intervention of interest" and which one was the "comparator".

	1	2	3	4	5	
subitem not at all important	○	○	○	○	◉	essential

Zrušit výběr

Does your paper address subitem 11a-ii?

Copy and paste relevant sections from the manuscript (include quotes in quotation marks "like this" to indicate direct quotes from your manuscript), or elaborate on this item by providing additional information not in the ms, or briefly explain why the item is not applicable/relevant for your study

No / Not fully.
The manuscript does not explicitly describe whether participants and their parents were informed which of the two interventions (intensive NEUROEQUIP-SMA vs. conventional physiotherapy) was considered the "intervention of interest" and which one was the "comparator."

11b) If relevant, description of the similarity of interventions
(this item is usually not relevant for ehealth trials as it refers to similarity of a placebo or sham intervention to a active medication/intervention)

Does your paper address CONSORT subitem 11b? *

Copy and paste relevant sections from the manuscript (include quotes in quotation marks "like this" to indicate direct quotes from your manuscript), or elaborate on this item by providing additional information not in the ms, or briefly explain why the item is not applicable/relevant for your study

Not applicable.
This study compares two active physiotherapeutic interventions (intensive NEUROEQUIP-SMA vs. conventional physiotherapy). There is no placebo, sham therapy, or inactive control condition. Because both interventions differ in content, duration, procedures, and therapeutic aims, it is neither possible nor relevant to match them for similarity. Therefore, this CONSORT item does not apply to this type of non-pharmacological trial.

12a) Statistical methods used to compare groups for primary and secondary outcomes

NPT: When applicable, details of whether and how the clustering by care providers or centers was addressed

Does your paper address CONSORT subitem 12a? *

Copy and paste relevant sections from the manuscript (include quotes in quotation marks "like this" to indicate direct quotes from your manuscript), or elaborate on this item by providing additional information not in the ms, or briefly explain why the item is not applicable/relevant for your study

Does your paper address CONSORT subitem 12a?

Yes.
The manuscript contains a detailed description of the statistical methods used to compare groups for primary and secondary outcomes. Relevant excerpts include:

"Statistical analysis and software
Data will be presented as means with standard .... quantitative outcome, the treatment effect will be evaluated by testing within-subject differences between NEUROEQUIP-SMA therapy (A) and standard therapy (B) using a paired t-test."

"If the normality assumption is not met, the exact Wilcoxon signed-rank test will be used instead of the paired t-test, and the exact Wilcoxon rank-sum test instead of the two-sample t-test. Normality will be assessed both graphically (e.g., Q–Q plot) and using the Shapiro–Wilk test."

"For qualitative outcomes, the effects of treatment, sequence, and period will be assessed using McNemar's test for within-subject differences and Fisher's exact test for between-subject differences."

"When conducting multiple comparisons, the achieved levels of significance (p-values) will be adjusted using a correction method, such as the Holm-Bonferroni method."

"Effect sizes will be reported alongside p-values to provide insight into the magnitude of observed changes. Sensitivity analyses for outliers will be included, comparing results with and without these subjects."

"Data analysis will be performed using R software. Missing data will not be imputed."

12a-i) Imputation techniques to deal with attrition / missing values

Imputation techniques to deal with attrition / missing values: Not all participants will use the intervention/comparator as intended and attrition is typically high in ehealth trials. Specify how participants who did not use the application or dropped out from the trial were treated in the statistical analysis (a complete case analysis is strongly discouraged, and simple imputation techniques such as LOCF may also be problematic [4]).

	1	2	3	4	5	
subitem not at all important	○	◉	○	○	○	essential

Zrušit výběr

Does your paper address subitem 12a-i? *

Copy and paste relevant sections from the manuscript (include quotes in quotation marks "like this" to indicate direct quotes from your manuscript), or elaborate on this item by providing additional information not in the ms, or briefly explain why the item is not applicable/relevant for your study

“Missing data will not be imputed.”

12b) Methods for additional analyses, such as subgroup analyses and adjusted analyses

Does your paper address CONSORT subitem 12b? *

Copy and paste relevant sections from the manuscript (include quotes in quotation marks "like this" to indicate direct quotes from your manuscript), or elaborate on this item by providing additional information not in the ms, or briefly explain why the item is not applicable/relevant for your study

No predefined subgroup analyses or adjusted analyses are planned. The manuscript states that the statistical analysis focuses on treatment, sequence, and period effects within the randomized crossover design, using paired and unpaired tests as appropriate. The only additional procedures described are sensitivity analyses for outliers, in which results will be compared with and without the outlier subjects to assess robustness. No further exploratory, subgroup, or adjusted analyses are planned.

Relevant manuscript text:

“Effect sizes will be reported alongside p-values to provide insight into the magnitude of observed changes. Sensitivity analyses for outliers will be included, comparing results with and without these subjects.”

“Not only the treatment effect, but also potential period and sequence effects on the outcomes will be tested; however, they will be analysed separately due to the small sample size.”

X26) REB/IRB Approval and Ethical Considerations [recommended as subheading under "Methods"] (not a CONSORT item)

X26-i) Comment on ethics committee approval

	1	2	3	4	5	
subitem not at all important	○	○	○	○	◉	essential

Zrušit výběr

Does your paper address subitem X26-i?

Copy and paste relevant sections from the manuscript (include quotes in quotation marks "like this" to indicate direct quotes from your manuscript), or elaborate on this item by providing additional information not in the ms, or briefly explain why the item is not applicable/relevant for your study

Yes.
The manuscript explicitly reports ethics committee approval:

"The multicentric ethics committee of the Third Medical Faculty of Charles University has approved the study under the code UK3LF/658559/12025, based on submitted informed consent forms."

x26-ii) Outline informed consent procedures

Outline informed consent procedures e.g., if consent was obtained offline or online (how? Checkbox, etc.?), and what information was provided (see 4a-ii). See [6] for some items to be included in informed consent documents.

	1	2	3	4	5	
subitem not at all important	○	○	○	○	◉	essential

Zrušit výběr

Does your paper address subitem X26-ii?

Copy and paste relevant sections from the manuscript (include quotes in quotation marks "like this" to indicate direct quotes from your manuscript), or elaborate on this item by providing additional information not in the ms, or briefly explain why the item is not applicable/relevant for your study

Yes.
The manuscript clearly outlines the informed consent procedures.

Relevant passages from the manuscript include:

"Since we are dealing with child participants, informed consent must be signed by parents or legal guardians."

X26-iii) Safety and security procedures

Safety and security procedures, incl. privacy considerations, and any steps taken to reduce the likelihood or detection of harm (e.g., education and training, availability of a hotline)

	1	2	3	4	5	
subitem not at all important	○	○	○	○	◉	essential

Zrušit výběr

Does your paper address subitem X26-iii?

Copy and paste relevant sections from the manuscript (include quotes in quotation marks "like this" to indicate direct quotes from your manuscript), or elaborate on this item by providing additional information not in the ms, or briefly explain why the item is not applicable/relevant for your study

"The horses performing the therapy in the study have specialized examinations, and the trainers are professionals with many years of experience to sufficiently ensure safety during NEUROEQUIP-SMA and therapeutic care."

RESULTS

13a) For each group, the numbers of participants who were randomly assigned, received intended treatment, and were analysed for the primary outcome

NPT: The number of care providers or centers performing the intervention in each group and the number of patients treated by each care provider in each center

Does your paper address CONSORT subitem 13a? *

Copy and paste relevant sections from the manuscript (include quotes in quotation marks "like this" to indicate direct quotes from your manuscript), or elaborate on this item by providing additional information not in the ms, or briefly explain why the item is not applicable/relevant for your study

Not applicable.
This manuscript reports a study protocol, and therefore no participants have yet been randomized, treated, or analysed.

13b) For each group, losses and exclusions after randomisation, together with reasons

Does your paper address CONSORT subitem 13b? (NOTE: Preferably, this is shown in a CONSORT flow diagram) *

Copy and paste relevant sections from the manuscript (include quotes in quotation marks "like this" to indicate direct quotes from your manuscript), or elaborate on this item by providing additional information not in the ms, or briefly explain why the item is not applicable/relevant for your study

Not applicable.
This manuscript describes a study protocol, and therefore no participants have yet been randomized or entered into the study.

13b-i) Attrition diagram

Strongly recommended: An attrition diagram (e.g., proportion of participants still logging in or using the intervention/comparator in each group plotted over time, similar to a survival curve) or other figures or tables demonstrating usage/dose/engagement.

	1	2	3	4	5	
subitem not at all important	○	○	◉	○	○	essential

Zrušit výběr

Does your paper address subitem 13b-i?

Copy and paste relevant sections from the manuscript or cite the figure number if applicable (include quotes in quotation marks "like this" to indicate direct quotes from your manuscript), or elaborate on this item by providing additional information not in the ms, or briefly explain why the item is not applicable/relevant for your study

Your study does not include any digital intervention, no logins, no usage logs, no dose–response through software platforms, and no online engagement data.

14a) Dates defining the periods of recruitment and follow-up

Does your paper address CONSORT subitem 14a? *

Copy and paste relevant sections from the manuscript (include quotes in quotation marks "like this" to indicate direct quotes from your manuscript), or elaborate on this item by providing additional information not in the ms, or briefly explain why the item is not applicable/relevant for your study

Yes.
Revised manuscript clearly reports (1) recruitment period, (2) data collection period, a (3) planned follow-up timeline.

14a-i) Indicate if critical “secular events” fell into the study period

Indicate if critical “secular events” fell into the study period, e.g., significant changes in Internet resources available or “changes in computer hardware or Internet delivery resources”

	1	2	3	4	5	
subitem not at all important	◉	○	○	○	○	essential

Zrušit výběr

Does your paper address subitem 14a-i?

Copy and paste relevant sections from the manuscript (include quotes in quotation marks "like this" to indicate direct quotes from your manuscript), or elaborate on this item by providing additional information not in the ms, or briefly explain why the item is not applicable/relevant for your study

No critical secular events occurred during the study period. The trial does not involve any digital, online, or software-based intervention components, so changes in computer hardware, software, or Internet resources are not relevant.

14b) Why the trial ended or was stopped (early)

Does your paper address CONSORT subitem 14b? *

Copy and paste relevant sections from the manuscript (include quotes in quotation marks "like this" to indicate direct quotes from your manuscript), or elaborate on this item by providing additional information not in the ms, or briefly explain why the item is not applicable/relevant for your study

Yes.Below is the relevant section from the manuscript:
"No interim analyses are planned. The trial will be conducted in full as described, unless terminated early for ethical or safety reasons upon recommendation of the principal investigator or the ethics committee."

15) A table showing baseline demographic and clinical characteristics for each group

NPT: When applicable, a description of care providers (case volume, qualification, expertise, etc.) and centers (volume) in each group

Does your paper address CONSORT subitem 15? *

Copy and paste relevant sections from the manuscript (include quotes in quotation marks "like this" to indicate direct quotes from your manuscript), or elaborate on this item by providing additional information not in the ms, or briefly explain why the item is not applicable/relevant for your study

No — baseline demographic and clinical characteristics are not presented in the manuscript, because this document is a study protocol, not a results paper.

15-i) Report demographics associated with digital divide issues

In ehealth trials it is particularly important to report demographics associated with digital divide issues, such as age, education, gender, social-economic status, computer/Internet/ehealth literacy of the participants, if known.

	1	2	3	4	5	
subitem not at all important	◉	○	○	○	○	essential

Zrušit výběr

Does your paper address subitem 15-i? *

Copy and paste relevant sections from the manuscript (include quotes in quotation marks "like this" to indicate direct quotes from your manuscript), or elaborate on this item by providing additional information not in the ms, or briefly explain why the item is not applicable/relevant for your study

This study does not include any digital, online, or eHealth intervention components. All interventions and assessments are delivered in person, and therefore demographics associated with digital divide issues (internet access, computer/technology literacy, or eHealth literacy) are not relevant and not reported.

16) For each group, number of participants (denominator) included in each analysis and whether the analysis was by original assigned groups

16-i) Report multiple “denominators” and provide definitions

Report multiple “denominators” and provide definitions: Report N’s (and effect sizes) “across a range of study participation [and use] thresholds” [1], e.g., N exposed, N consented, N used more than x times, N used more than y weeks, N participants “used” the intervention/comparator at specific pre-defined time points of interest (in absolute and relative numbers per group). Always clearly define “use” of the intervention.

	1	2	3	4	5	
subitem not at all important	◉	○	○	○	○	essential

Zrušit výběr

Does your paper address subitem 16-i? *

Copy and paste relevant sections from the manuscript (include quotes in quotation marks "like this" to indicate direct quotes from your manuscript), or elaborate on this item by providing additional information not in the ms, or briefly explain why the item is not applicable/relevant for your study

No — this subitem is not applicable because the study does not include any digital “use” metrics and no analyses have yet been performed (protocol stage).

16-ii) Primary analysis should be intent-to-treat

Primary analysis should be intent-to-treat, secondary analyses could include comparing only “users”, with the appropriate caveats that this is no longer a randomized sample (see 18-i).

	1	2	3	4	5	
subitem not at all important	○	◉	○	○	○	essential

Zrušit výběr

Does your paper address subitem 16-ii?

Copy and paste relevant sections from the manuscript (include quotes in quotation marks "like this" to indicate direct quotes from your manuscript), or elaborate on this item by providing additional information not in the ms, or briefly explain why the item is not applicable/relevant for your study

The manuscript does not explicitly state whether the primary analysis will follow an intent-to-treat (ITT) approach. As this is a protocol, no analyses have yet been performed.

17a) For each primary and secondary outcome, results for each group, and the estimated effect size and its precision (such as 95% confidence interval)

Does your paper address CONSORT subitem 17a? *

Copy and paste relevant sections from the manuscript (include quotes in quotation marks "like this" to indicate direct quotes from your manuscript), or elaborate on this item by providing additional information not in the ms, or briefly explain why the item is not applicable/relevant for your study

No — this item is not applicable to the current manuscript, because this is a study protocol and no results have yet been collected or analyzed.

17a-i) Presentation of process outcomes such as metrics of use and intensity of use

In addition to primary/secondary (clinical) outcomes, the presentation of process outcomes such as metrics of use and intensity of use (dose, exposure) and their operational definitions is critical. This does not only refer to metrics of attrition (13-b) (often a binary variable), but also to more continuous exposure metrics such as “average session length”. These must be accompanied by a technical description how a metric like a “session” is defined (e.g., timeout after idle time) [1] (report under item 6a).

	1	2	3	4	5	
subitem not at all important	◉	○	○	○	○	essential

Zrušit výběr

Does your paper address subitem 17a-i?

Copy and paste relevant sections from the manuscript (include quotes in quotation marks "like this" to indicate direct quotes from your manuscript), or elaborate on this item by providing additional information not in the ms, or briefly explain why the item is not applicable/relevant for your study

Not applicable — the study does not involve any digital system, app, platform, or online component that would generate “use”, “session length”, “logins”, or other eHealth exposure metrics.

17b) For binary outcomes, presentation of both absolute and relative effect sizes is recommended

Does your paper address CONSORT subitem 17b? *

Copy and paste relevant sections from the manuscript (include quotes in quotation marks "like this" to indicate direct quotes from your manuscript), or elaborate on this item by providing additional information not in the ms, or briefly explain why the item is not applicable/relevant for your study

At this stage the protocol does not report binary outcome results, because no results are yet available.

18) Results of any other analyses performed, including subgroup analyses and adjusted analyses, distinguishing pre-specified from exploratory

Does your paper address CONSORT subitem 18? *

Copy and paste relevant sections from the manuscript (include quotes in quotation marks "like this" to indicate direct quotes from your manuscript), or elaborate on this item by providing additional information not in the ms, or briefly explain why the item is not applicable/relevant for your study

No — this item is not applicable because the manuscript is a study protocol and no analyses (including subgroup or adjusted analyses) have been conducted yet.

18-i) Subgroup analysis of comparing only users

A subgroup analysis of comparing only users is not uncommon in ehealth trials, but if done, it must be stressed that this is a self-selected sample and no longer an unbiased sample from a randomized trial (see 16-iii).

	1	2	3	4	5	
subitem not at all important	◉	○	○	○	○	essential

Zrušit výběr

Does your paper address subitem 18-i?

Copy and paste relevant sections from the manuscript (include quotes in quotation marks "like this" to indicate direct quotes from your manuscript), or elaborate on this item by providing additional information not in the ms, or briefly explain why the item is not applicable/relevant for your study

No — this subitem is not applicable because the study does not involve any digital intervention, online platform, or “users versus non-users” distinction.

19) All important harms or unintended effects in each group

(for specific guidance see CONSORT for harms)

Does your paper address CONSORT subitem 19? *

Copy and paste relevant sections from the manuscript (include quotes in quotation marks "like this" to indicate direct quotes from your manuscript), or elaborate on this item by providing additional information not in the ms, or briefly explain why the item is not applicable/relevant for your study

Yes — the manuscript addresses this item by describing how harms, adverse events, and safety concerns will be monitored.
Because this is a study protocol, no actual harms are yet available, but the protocol clearly specifies procedures for detecting, reporting, and responding to adverse events.
"All adverse events must be reported to the principal investigator and the appropriate specialist. All adverse events (AEs) will be monitored and systematically recorded by the treating physiotherapists during the study using a predefined reporting form."

19-i) Include privacy breaches, technical problems

Include privacy breaches, technical problems. This does not only include physical "harm" to participants, but also incidents such as perceived or real privacy breaches [1], technical problems, and other unexpected/unintended incidents. "Unintended effects" also includes unintended positive effects [2].

	1	2	3	4	5	
subitem not at all important	(●)	( )	( )	( )	( )	essential

Zrušit výběr

Does your paper address subitem 19-i?

Copy and paste relevant sections from the manuscript (include quotes in quotation marks "like this" to indicate direct quotes from your manuscript), or elaborate on this item by providing additional information not in the ms, or briefly explain why the item is not applicable/relevant for your study

No — this subitem is not applicable because the study does not involve any digital systems, online data collection, electronic platforms, or technologies that could generate privacy breaches or technical problems.

19-ii) Include qualitative feedback from participants or observations from staff/researchers

Include qualitative feedback from participants or observations from staff/researchers, if available, on strengths and shortcomings of the application, especially if they point to unintended/unexpected effects or uses. This includes (if available) reasons for why people did or did not use the application as intended by the developers.

	1	2	3	4	5	
subitem not at all important	(●)	( )	( )	( )	( )	essential

Zrušit výběr

Does your paper address subitem 19-ii?

Copy and paste relevant sections from the manuscript (include quotes in quotation marks "like this" to indicate direct quotes from your manuscript), or elaborate on this item by providing additional information not in the ms, or briefly explain why the item is not applicable/relevant for your study

No — this subitem is not applicable to the present study protocol.
The manuscript does not include any qualitative feedback from participants or staff because no participants have yet been enrolled.

DISCUSSION

22) Interpretation consistent with results, balancing benefits and harms, and considering other relevant evidence

NPT: In addition, take into account the choice of the comparator, lack of or partial blinding, and unequal expertise of care providers or centers in each group

22-i) Restate study questions and summarize the answers suggested by the data, starting with primary outcomes and process outcomes (use)

Restate study questions and summarize the answers suggested by the data, starting with primary outcomes and process outcomes (use).

	1	2	3	4	5	
subitem not at all important	(•)	( )	( )	( )	( )	essential

Zrušit výběr

Does your paper address subitem 22-i? *

Copy and paste relevant sections from the manuscript (include quotes in quotation marks "like this" to indicate direct quotes from your manuscript), or elaborate on this item by providing additional information not in the ms, or briefly explain why the item is not applicable/relevant for your study

No — this item is not applicable because the manuscript is a study protocol and therefore contains no results to interpret.

22-ii) Highlight unanswered new questions, suggest future research

Highlight unanswered new questions, suggest future research.

	1	2	3	4	5	
subitem not at all important	( )	( )	(•)	( )	( )	essential

Zrušit výběr

Does your paper address subitem 22-ii?

Copy and paste relevant sections from the manuscript (include quotes in quotation marks "like this" to indicate direct quotes from your manuscript), or elaborate on this item by providing additional information not in the ms, or briefly explain why the item is not applicable/relevant for your study

The manuscript clearly sets out unanswered questions and suggests future research directions.
“Research to better elucidate the intensity of physiotherapy has not yet been published.”

20) Trial limitations, addressing sources of potential bias, imprecision, and, if relevant, multiplicity of analyses

20-i) Typical limitations in ehealth trials

Typical limitations in ehealth trials: Participants in ehealth trials are rarely blinded. Ehealth trials often look at a multiplicity of outcomes, increasing risk for a Type I error. Discuss biases due to non-use of the intervention/usability issues, biases through informed consent procedures, unexpected events.

	1	2	3	4	5	
subitem not at all important	(•)	( )	( )	( )	( )	essential

Zrušit výběr

Does your paper address subitem 20-i? *

Copy and paste relevant sections from the manuscript (include quotes in quotation marks "like this" to indicate direct quotes from your manuscript), or elaborate on this item by providing additional information not in the ms, or briefly explain why the item is not applicable/relevant for your study

No — this subitem is not applicable, because the study does not involve any eHealth, digital, or online intervention.

21) Generalisability (external validity, applicability) of the trial findings

NPT: External validity of the trial findings according to the intervention, comparators, patients, and care providers or centers involved in the trial

21-i) Generalizability to other populations

Generalizability to other populations: In particular, discuss generalizability to a general Internet population, outside of a RCT setting, and general patient population, including applicability of the study results for other organizations

	1	2	3	4	5	
subitem not at all important	○	◉	○	○	○	essential

Zrušit výběr

Does your paper address subitem 21-i?

Copy and paste relevant sections from the manuscript (include quotes in quotation marks "like this" to indicate direct quotes from your manuscript), or elaborate on this item by providing additional information not in the ms, or briefly explain why the item is not applicable/relevant for your study

Partially. This study does not include any digital or Internet-based intervention; therefore, generalizability to the ‘general Internet population’ is not applicable. The manuscript does describe characteristics of the intervention settings and care providers, which supports assessment of external validity in clinical contexts, but it does not and cannot address generalizability to online populations. As a protocol, no results are yet available to discuss broader applicability.

21-ii) Discuss if there were elements in the RCT that would be different in a routine application setting

Discuss if there were elements in the RCT that would be different in a routine application setting (e.g., prompts/reminders, more human involvement, training sessions or other co-interventions) and what impact the omission of these elements could have on use, adoption, or outcomes if the intervention is applied outside of a RCT setting.

	1	2	3	4	5	
subitem not at all important	○	○	◉	○	○	essential

Zrušit výběr

Does your paper address subitem 21-ii?

Copy and paste relevant sections from the manuscript (include quotes in quotation marks "like this" to indicate direct quotes from your manuscript), or elaborate on this item by providing additional information not in the ms, or briefly explain why the item is not applicable/relevant for your study

Partially. This study does not include any digital or eHealth intervention; therefore, elements such as prompts, digital reminders, training for application use, or adoption differences outside an RCT setting are not applicable. The intervention is entirely therapist-delivered in person. The manuscript describes the qualifications of the physiotherapists and the characteristics of the therapeutic setting, which supports assessment of clinical generalizability, but it does not explicitly discuss how RCT-specific elements (e.g., structured schedules, intensive daily sessions, controlled staffing) may differ from routine clinical practice.

OTHER INFORMATION

23) Registration number and name of trial registry

Does your paper address CONSORT subitem 23? *

Copy and paste relevant sections from the manuscript (include quotes in quotation marks "like this" to indicate direct quotes from your manuscript), or elaborate on this item by providing additional information not in the ms, or briefly explain why the item is not applicable/relevant for your study

The trial has been submitted for registration in ClinicalTrials.gov, but the registration number has not yet been issued at the time of manuscript submission. The registry name (ClinicalTrials.gov) is reported in the manuscript, and the registration number will be added once assigned.

24) Where the full trial protocol can be accessed, if available

Does your paper address CONSORT subitem 24? *

Cite a Multimedia Appendix, other reference, or copy and paste relevant sections from the manuscript (include quotes in quotation marks "like this" to indicate direct quotes from your manuscript), or elaborate on this item by providing additional information not in the ms, or briefly explain why the item is not applicable/relevant for your study

Partially — the full protocol is provided directly within the manuscript, but it is not yet publicly accessible in any external registry or repository.

25) Sources of funding and other support (such as supply of drugs), role of funders

Does your paper address CONSORT subitem 25? *

Copy and paste relevant sections from the manuscript (include quotes in quotation marks "like this" to indicate direct quotes from your manuscript), or elaborate on this item by providing additional information not in the ms, or briefly explain why the item is not applicable/relevant for your study

Yes. Relevant section from the manuscript:
“The study is funded by the Grant Agency of Charles University (4424; January 2024), the Cooperatio program (Neurosciences) and SVV 260533/SVV/2024.”

X27) Conflicts of Interest (not a CONSORT item)

X27-i) State the relation of the study team towards the system being evaluated

In addition to the usual declaration of interests (financial or otherwise), also state the relation of the study team towards the system being evaluated, i.e., state if the authors/evaluators are distinct from or identical with the developers/sponsors of the intervention.

	1	2	3	4	5	
subitem not at all important	○	○	○	○	◉	essential

Zrušit výběr

Does your paper address subitem X27-i?

Copy and paste relevant sections from the manuscript (include quotes in quotation marks "like this" to indicate direct quotes from your manuscript), or elaborate on this item by providing additional information not in the ms, or briefly explain why the item is not applicable/relevant for your study

Yes, the manuscript addresses conflicts of interest, including the relationship of the study team to the evaluated system. The relevant section states:

"The authors declare that the research was conducted in the absence of any commercial or financial relationships that could be construed as a potential conflict of interest."

About the CONSORT EHEALTH checklist

As a result of using this checklist, did you make changes in your manuscript? *

- ○ yes, major changes
- ◉ yes, minor changes
- ○ no

What were the most important changes you made as a result of using this checklist?

The checklist also led us to clarify and strengthen our description of blinding. We added that parents will not be informed which of the two therapies is considered the ‘intervention of interest’ and which one is the comparator. During consent and throughout the study, parents and children will only be told that the child will receive two different physiotherapy approaches based on the same neuroproprioceptive principles.

How much time did you spend on going through the checklist INCLUDING making changes in your manuscript *

5 hours

Your answer must have a minimum of 25 characters.

As a result of using this checklist, do you think your manuscript has improved? *

- (x) yes
- ( ) no
- ( ) Jiné:

Would you like to become involved in the CONSORT EHEALTH group?

This would involve for example becoming involved in participating in a workshop and writing an "Explanation and Elaboration" document

- (x) yes
- ( ) no
- ( ) Jiné:

Zrušit výběr

Any other comments or questions on CONSORT EHEALTH

Vaše odpověď

STOP - Save this form as PDF before you click submit

To generate a record that you filled in this form, we recommend to generate a PDF of this page (on a Mac, simply select "print" and then select "print as PDF") before you submit it.

When you submit your (revised) paper to JMIR, please upload the PDF as supplementary file.

Don't worry if some text in the textboxes is cut off, as we still have the complete information in our database. Thank you!

Final step: Click submit !

Click submit so we have your answers in our database!

Odeslat Vymazat formulář

Nikdy přes Formuláře Google neposílejte hesla.

Obsah není vytvořen ani schválen Googlem. - Vlastník kontaktního formuláře - Smluvní podmínky služby - Zásady ochrany soukromí

Vypadá tento formulář podezřele? Přehled

Google Formuláře
